# MicroRNAs in atrial fibrillation target genes in structural remodelling

**DOI:** 10.1007/s00441-023-03823-0

**Published:** 2023-10-14

**Authors:** Nicoline W. E. van den Berg, Makiri Kawasaki, Fransisca A. Nariswari, Benedetta Fabrizi, Jolien Neefs, Ingeborg van der Made, Robin Wesselink, Wim Jan P. van Boven, Antoine H. G. Driessen, Aldo Jongejan, Joris R. de Groot

**Affiliations:** 1 Amsterdam UMC, University of Amsterdam, Heart Center; Department of Clinical and Experimental Cardiology and Cardiothoracic Surgery, Amsterdam Cardiovascular Sciences, Meibergdreef 9, 1105AZ Amsterdam, The Netherlands; 2 Amsterdam UMC, Department of Epidemiology and Data Science, University of Amsterdam, Meibergdreef 9, 1105AZ Amsterdam, The Netherlands

**Keywords:** Atrial fibrillation, MicroRNA, Transcriptome sequencing, Structural remodelling, Fibrosis

## Abstract

We aim to elucidate how miRNAs regulate the mRNA signature of atrial fibrillation (AF), to gain mechanistic insight and identify candidate targets for future therapies. We present combined miRNA–mRNA sequencing using atrial tissues of patient without AF (*n* = 22), with paroxysmal AF (*n* = 22) and with persistent AF (*n* = 20). mRNA sequencing previously uncovered upregulated epithelial to mesenchymal transition, endothelial cell proliferation and extracellular matrix remodelling involving glycoproteins and proteoglycans in AF. MiRNA co-sequencing discovered miRNAs regulating the mRNA expression changes. Key downregulated miRNAs included miR-135b-5p, miR-138-5p, miR-200a-3p, miR-200b-3p and miR-31-5p and key upregulated miRNAs were miR-144-3p, miR-15b-3p, miR-182-5p miR-18b-5p, miR-4306 and miR-206. MiRNA expression levels were negatively correlated with the expression levels of a multitude of predicted target genes. Downregulated miRNAs associated with increased gene expression are involved in upregulated epithelial and endothelial cell migration and glycosaminoglycan biosynthesis. In vitro inhibition of miR-135b-5p and miR-138-5p validated an effect of miRNAs on multiple predicted targets. Altogether, the discovered miRNAs may be explored in further functional studies as potential targets for anti-fibrotic therapies in AF.

**Supplementary Information:**

The online version contains supplementary material available at 10.1007/s00441-023-03823-0.

## Introduction

Atrial fibrillation (AF) is the most common cardiac arrhythmia, and as its prevalence increases, it is increasingly becoming an important health care issue (Krijthe et al. 2013; Chugh et al. 2014). AF is accompanied by a reduced quality of life, an increased stroke risk and doubled mortality rate (Hindricks et al. 2020). Currently, employed drug therapies primarily aim to restore sinus rhythm or to prevent stroke, but no contemporary AF therapy aims to reversely remodel or prevent progressive remodelling of the atrial substrate (Hindricks et al. 2020). Structural remodelling is a key remodelling process in AF pathophysiology and is characterized by atrial fibrosis (Nattel et al. 2020). The identification of novel candidates for fibrosis-targeted therapies requires an improved understanding of AF structural remodelling and the identification of most relevant regulators.

Atrial fibrosis is the result of complex interactions between various signalling pathways and activated (myo)fibroblasts that deposit an excess and altered extracellular matrix (ECM) (Nattel and Harada 2014; Dzeshka et al. 2015). We recently reported mRNA sequencing results demonstrating that altered ECM deposits include proteoglycans and glycoproteins and are associated with epithelial to mesenchymal transition (EMT) and endothelial cell proliferation, differentiation and migration. EMT signified an atrial-wide activation of (myo)fibroblast-like cells (Zeisberg et al. 2007; Suffee et al. 2020; Berg et al. 2021).

MiRNAs are key posttranscriptional mediators of gene expression that are thought to modulate the expression of ~ 50% of protein-coding genes including genes of structural remodelling (Friedman et al. 2009). MiRNAs are short, non-coding RNAs that mostly bind to the 3′-untranslated region (3′UTR) of the mRNA and thereby initiate degradation or translation repression (Bartel 2009). Because of their key regulatory role and ability to target multiple targets, miRNAs have the potential to modify a whole disease phenotype (Chakraborty et al. 2021). MiRNAs can therefore provide valuable complementary information on the regulation of the AF mRNA signature and have emerged as promising candidates for future therapies using synthetic miRNA mimics and inhibitors. The first miRNA therapeutic for the treatment of humane hepatitis C virus is currently in phase II clinical trials, underscoring the potential of miRNA-based therapy (Janssen et al. 2013; Chakraborty et al. 2021).

There is limited information on the relation between altered miRNA regulation and AF, as discrepancies in the direction that miRNAs change in AF remain, dependent on the material studied (van den Berg et al. 2017). Furthermore, miRNA discovery studies alone provide no information on the transcriptional effects and possible biological implications of altered miRNAs in AF pathophysiology. On the other hand, funtional studies have demonstrated a role for miRNAs in various processes such as inflammation, oxidative stress and ECM remodelling in AF (van den Berg et al. 2017), but our knowledge remains fragmented as most studies concentrate on a small number of miRNAs (targets) at a time.

MiRNA and mRNA co-sequencing may add biological significance to discovered miRNAs (van Iterson et al. 2013) and has only been performed in AF studies that focus on one particular pathway (Wang et al. 2015) or included patients with mitral valve disease (Larupa Santos et al. 2020). We combined miRNA sequencing with mRNA sequencing in a large and clinically well profiled cohort of patients undergoing mostly coronary artery bypass grafting without AF, and patients with paroxysmal AF and with persistent AF undergoing thoracoscopic AF ablation. Hereby, we determined broad regulatory miRNA effects on mRNA signatures in AF and uncovered potential miRNA functional implications (van Iterson et al. 2013).

## Materials and methods

### Study design and study population

**Fig. 1:**
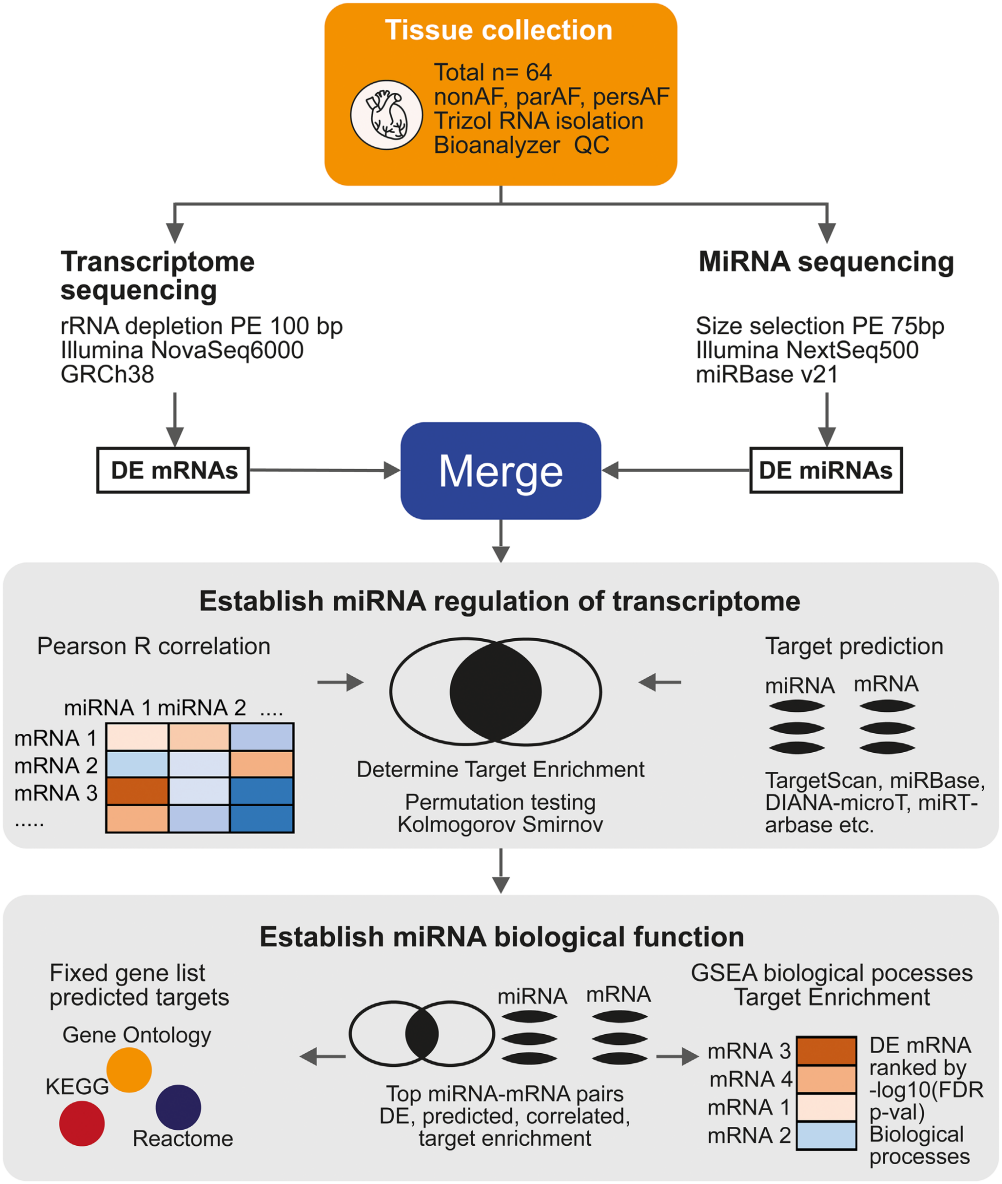
Study design. MiRNA and mRNA sequencing were independently performed in atrial tissues from the same patients. Results of the mRNA sequencing were reported previously. Analysed datasets of miRNAs and mRNAs were combined on the basis of target - prediction algorithms (online databases) and Pearson *R* correlations. Permutation testing and Kolmogorov–Smirnov tests were used to statistically establish a miRNA effect on mRNA signatures. Secondly, correlated and predicted targets were used for gene set enrichment (gProfiler). Inversely, permutation testing was used to determine whether the dysregulated biological processes discovered by the RNA sequencing, were regulated by differentially expressed miRNAs

We performed transcriptome sequencing of atrial tissues comparing patients without AF (nonAF, *n* = 22), patients with paroxysmal AF (parAF, *n* = 22) and patients with persistent AF (persAF, (*n* = 20, pooled with 2 longstanding persAF). The differential mRNA profiles of patients with and without AF were previously reported (Berg et al. 2021). In the current study, we performed miRNA sequencing in the same set of RNA samples. MiRNA function was assessed by combining the miRNA and mRNA sequencing data (Fig. 1).

Control subjects without a history of AF were included in the PREDICT-AF study (NCT03130985) (van den Berg et al. 2019; de Groot et al. 2020). The design of the PREDICT-AF study has been described previously. For ethical reasons, the study included 150 patients undergoing cardiothoracic surgery, mostly coronary artery bypass grafting, with an increased stroke risk defined by a CHA_2_DS_2_-VASc score of ≥ 2. Patients with AF were included in the AFACT trial (NCT01091389) and MARK AF registry (Driessen et al. 2016). Both studies applied the same inclusion and exclusion criteria to select patients with parAF or persAF undergoing thoracoscopic AF ablation. The left atrial appendage (LAA) was removed in all patients and stored in the ADAPT biobank (NCT04776642) for the discovery of biomarkers. ParAF, persAF and longstanding persAF were defined according to current guidelines (Hindricks et al. 2020).

All subjects were thoroughly clinically characterized, underwent pre-operative screening and echocardiography prior to surgery. Tissue removal and tissue storage were conducted in accordance with the protocol. The study was approved by both the Institutional Review Board and the Biobank review board of the Amsterdam University Medical Centre location AMC and was in accordance with the Declaration of Helsinki. All patients provided written informed consent.

### Tissue collection and RNA extraction

Upon excision, LAAs were immediately washed in ice-cold modified Tyrode’s solution before one-half was fixated in formaldehyde and the other half was snap-frozen in liquid nitrogen in the operating theatre and stored at − 80 °C. We extracted RNA from 50–100 mg of snap-frozen LAA whole tissues using Trizol (Invitrogen™, cat. no. 15596018). Samples were used for whole transcriptome sequencing, the quality of which was confirmed by bioanalyzer values (RIN values 8.4 ± 0.9; Agilent 2100, CA, USA). Libraries for whole transcriptome sequencing and miRNA sequencing were prepared from the same pool of RNA.

### MRNA and miRNA sequencing

For whole transcriptome sequencing, RNA samples were rRNA-depleted and sequenced (paired-end, 100 bp) on the Illumina NovaSeq 6000 platform (Illumina, CA, USA). Reads were mapped to the human reference genome (GRCh38) with HiSAT2 v2.1.0 and counted with HTSeq v0.11 (see Data Supplement for details).

For miRNA deep sequencing, strand-specific cDNA libraries were constructed from size-selected RNA fragments followed by Illumina single-end sequencing (75 bp) on the Illumina NextSeq 500 sequencing system (Illumina, CA, USA). MiRNA expression data was analysed using the QuickMIRSeq pipeline (Zhao et al. 2017). Reads were initially mapped against miRBase V21 to generate count data and reports were updated to miRBase V22. After the multidimensional scaling, one nonAF sample showing the lowest complexity of the miRNA profile was excluded from further analysis (Fig. S1 in Data Supplement).

### Gene and miRNA differential expression analyses

For both miRNA and transcriptome sequencing data, a linear model was fit after variance stabilization (Voom, R package *Limma*) which was used to compute fold changes and false discovery rate (FDR) and adjusted *P*-values (Benjamini-Hochberg). Linear regression analyses were age- and gender-corrected. For further analyses of the transcriptome data, we selected protein-coding genes only.

A FDR *P* < 0.05 for any of the comparisons parAF vs. nonAF, persAF vs. parAF or persAF vs. nonAF was considered to represent significant differential expression (DE). DE miRNAs were selected for qPCR validation based on an absolute log2FC > 1 and log2 counts per million (CPM) > 1. This selection of top DE miRNAs was also used for an in-depth analysis of the miRNA effects on mRNA expression and to assess potential biological implications (details below).

### Assessment of miRNA effects on target gene expression

A miRNA–mRNA target database was constructed for all miRNAs and protein-coding mRNAs detected in the atrial tissues. We combined miRNA–mRNA interaction data from online databases that hold predicted targets (miRDB (Chen and Wang 2020) score ≥ 75; TargetScan (Agarwal et al. 2015) total context score ≥ − 0.30; DIANA-microT (Paraskevopoulou et al. 2013) score ≥ 0.85; RNA22 (Miranda et al. 2006) score ≥ 0.02 and free energy ≤ − 18) or validated targets (miRTarBase (Hsu et al. 2011), DIANA-TarBase (Vlachos et al. 2015)) (Details in Supplementary Materials and Supplementary Table S2). A predicted miRNA–mRNA interaction was defined as a predicted miRNA–mRNA pair by at least two out of four prediction databases or a pair predicted by one of the prediction databases and at least one of the validated databases.

Potential miRNA–mRNA interactions were discovered by computing Pearson *R* correlation coefficients for the DE miRNAs (*R* < − 0.4 and FDR < 0.05 (Benjamini-Hochberg)). A direct interaction for a miRNA–mRNA pair was assumed when target prediction overlaid with a negative correlation. With permutation testing (1000 ×), we established for the DE miRNAs whether predicted miRNA–mRNA interactions were more common among the negatively correlated miRNA–mRNA pairs than expected by chance (details in Supplementary Materials) (Okada et al. 2016).

For individual top DE miRNAs (log2FC > 1, CPM > 1), the Kolmogorov–Smirnov test was used to determine if negative miRNA–mRNA correlations were more common among predicted targets than non-predicted targets. Hereto, mRNAs were filtered based on CPM > 3 as an effect may only be detectable for genes with higher expression levels (Wang et al. 2019). For the applied thresholds in this study, multiple cut-offs were evaluated. Cut-offs were set for their discriminative abilities defined by enrichment scores, or number of discoveries reaching significance thresholds.

### Assessment of miRNA functional implications

Since multiple miRNAs may function synergistically with various combinations of their predicted targets, we determined whether miRNAs regulated the biological processes discovered in association with AF structural remodelling. Two hundred seventy biological processes that had previously been discovered by RNA sequencing (mRNA expression (sign(log2FC) * − log10(FDR)) (Berg et al. 2021) were assessed for an overrepresentation of DE miRNAs that are predicted to regulate genes from those gene sets using permutation testing (1000 ×) (details in Supplementary Materials).

As a complementary and more independent strategy, the predicted and correlated targets of the upregulated and downregulated miRNAs were used for gene set enrichment using gProfiler (gene ontology: biological processes, cellular component, molecular function, KEGG) (Raudvere et al. 2019).

For each analysis, an interaction network of the gene ontology biological processes was visualized with the Cytoscape plugin EnrichmentMap. MiRNAs and mRNAs presented in the figures were selected for their key roles, defined by their functional implications or frequency of occurrence as a predicted target.

### qPCR validation of miRNA and mRNA expression

For miRNA quantification, cDNA was synthesized with qScript cDNA SuperMix from 500 ng of total RNA from the same patients as used for sequencing (Quanta Biosciences). Real-time PCR was performed using PerfeCTa SYBR Green FastMix (Quanta Biosciences)*.*

For gene expression quantification, cDNA was synthesized from 500 ng of total RNA with SuperScript™ II reverse transcriptase (Invitrogen™, cat no. 18064022) and real-time PCR quantification was performed with the SYBR green PCR kit (Roche, cat. no. 04707516001). All experiments were performed in duplicate on the LightCycler480 (Roche).

Starting concentrations of each gene or miRNA were calculated using LinRegPCR (Ruijter et al. 2009). Values were normalized against the geometric mean of miR-27a-3p, miR-191-5p and miR-let7a-5p for miRNA expression and *GUSB*, *HPRT1* and *PGK1* for human gene expression and *Hprt* for rat. Normalizers were selected based upon literature and a lack of variability and high expression in the sequencing data (Peltier and Latham 2008; Masè et al. 2017; Berg et al. 2021). Primers used are displayed in Table S3 and Table S4 of the Data Supplement.

### Functional validation of discovered miRNAs

To inhibit endogenous miRNAs of interest, rat non-cardiomyocyte mesenchymal cells at approximately 70% confluency were transfected with anti-miR™ miRNA inhibitors (ThermoFisher Scientific, cat. no. AM17000) of miR-135b-5p at 80 nM (ThermoFisher Scientific, assay id AM13044), and of miR-138-5p at 20 nM (ThermoFisher Scientific, assay id AM11727) along with the corresponding amount of negative control (ThermoFisher Scientific, #AM17010) using Lipofectamine RNAiMax (ThermoFisher Scientific, #13778150). A detailed description of the neonatal rat ventricular non-cardiomyocytes isolation is available in the Data Supplement. Rat non-cardiomyocyte mesenchymal cells were cultured for 48 h before total RNA was extracted from each sample. The expression of their target genes was assessed by qPCR.

#### Statistical analysis

qPCR data were compared with ANOVA, Kruskal–Wallis, *T*-test or Mann–Whitney *U* tests, when appropriate. We compared nonAF, parAF and persAF patient groups. All performed tests were two-sided and a *P*-value < 0.05 was considered statistically significant (R version 3.2.3).

## Results

### Differential expressed miRNAs identify nonAF, paroxysmal and persistent AF patients

Sixty-four patients undergoing cardiac surgery were included (Fig. 1). These included patients participating in the PREDICT-AF trial, who had no history of AF (nonAF; *n* = 22), had a CHA_2_DS_2_-VASc ≥ 2 and underwent cardiac surgery, mostly coronary artery bypass grafting. Besides, patients with paroxysmal AF (parAF; *n* = 22) and with persistent AF (persAF; *n* = 20) undergoing AF ablation surgery were included. Due to the differences in surgery type and inclusion criteria for participation in the PREDICT-AF trial, control patients more frequently had a higher CHA_2_DS_2_-VASc score due to a history of coronary artery disease. Clinical characteristics of the study groups are displayed in Supplementary Table S1.

The atrial tissues from nonAF, parAF and persAF patients were used for mRNA and miRNA sequencing (Fig. 1). mRNA sequencing identified 5228 differentially expressed protein-coding genes involved in upregulated EMT, endothelial cell proliferation and ECM remodelling involving glycoproteins and proteoglycan synthesis. A detailed description of the mRNA sequencing with a quantitative and histological validation was described previously (Berg et al. 2021).

**Fig. 2:**
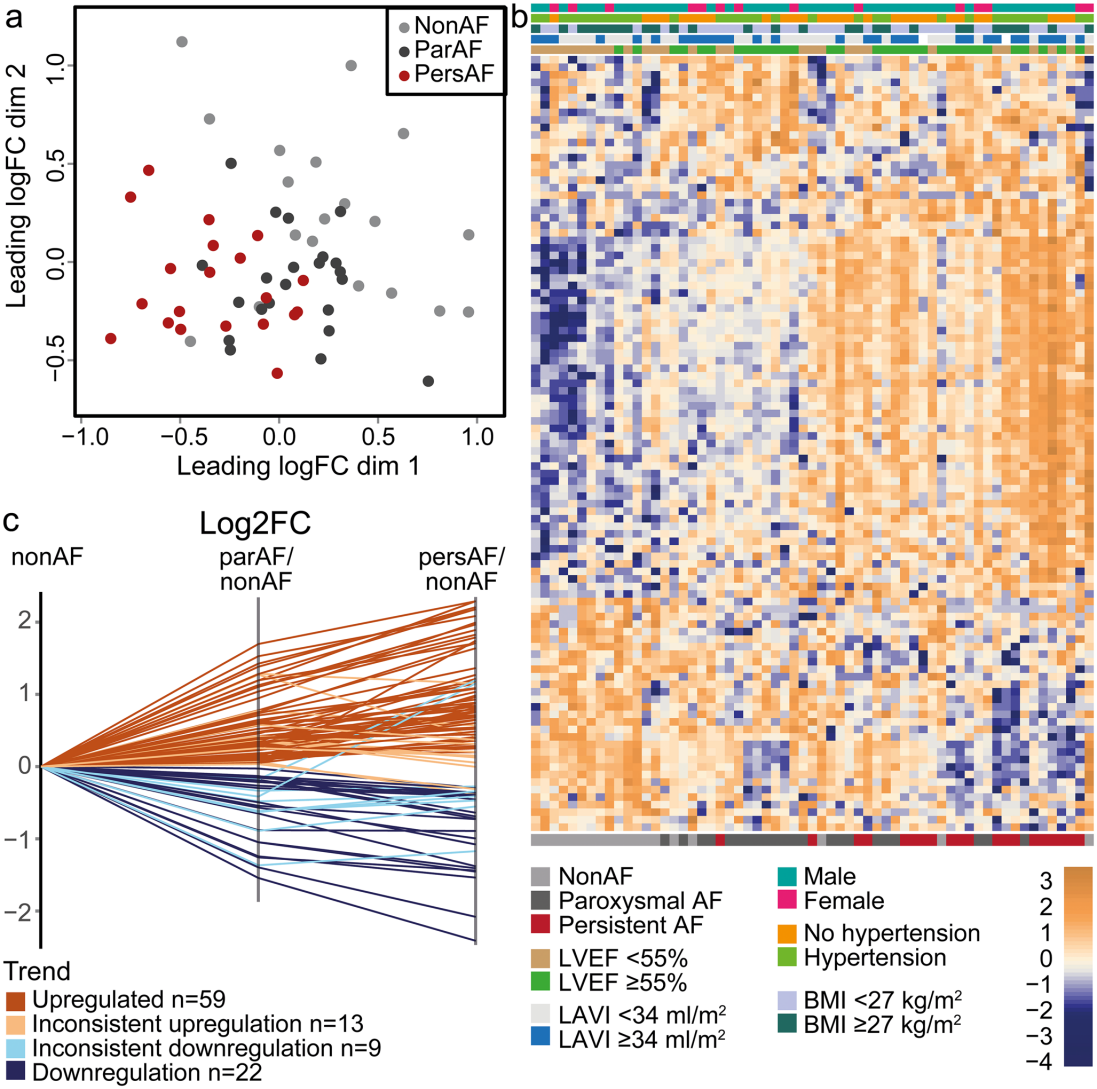
MiRNA expression signatures identify nonAF, parAF and persAF patients. **a** Dimensionality reduction shows a reasonable separation of nonAF, parAF and persAF patients. **b** Heatmap shows 103 miRNA that were DE between any of the three comparisons. Hierarchical clustering tends to separate nonAF from parAF and persAF patients. Patients show no clustering based on clinical characteristics or comorbidities. Most miRNAs demonstrate a consistent upward or downward trend from nonAF to parAF to persAF. **c** Log2FCs were plotted for 103 DE miRNAs using nonAF expression as reference. The graph illustrates the continuous expression trend seen in 81 of 103 DE miRNAs

The current study focuses on the discovery of miRNAs regulating the mRNA signature (Fig. 1). MiRNA sequencing identified 857 unique miRNAs expressed in the left atrium. Dimensionality reduction using multidimensional scaling showed a tended separation of nonAF, parAF and persAF study groups (Fig. 2a; Supplementary Fig. S1). There were 103 miRNAs DE between any of the three comparisons, that is, persAF vs. nonAF, parAF vs. nonAF or persAF vs. parAF (FDR *P* < 0.05). Unsupervised hierarchical clustering of patients and DE miRNAs largely separated the patients based on AF type and did not identify any clustering by clinical characteristics or comorbidities in the study cohort (Fig. 2b). The expression of the vast majority of DE miRNAs (79%) was continuously increased or decreased between nonAF, parAF and persAF (Fig. 2c). This ordinal trend of miRNA expression resembled the previously reported ordinal trend of mRNA expression between nonAF, parAF and persAF and suggests validity of the control group (Berg et al. 2021).

**Fig. 3:**
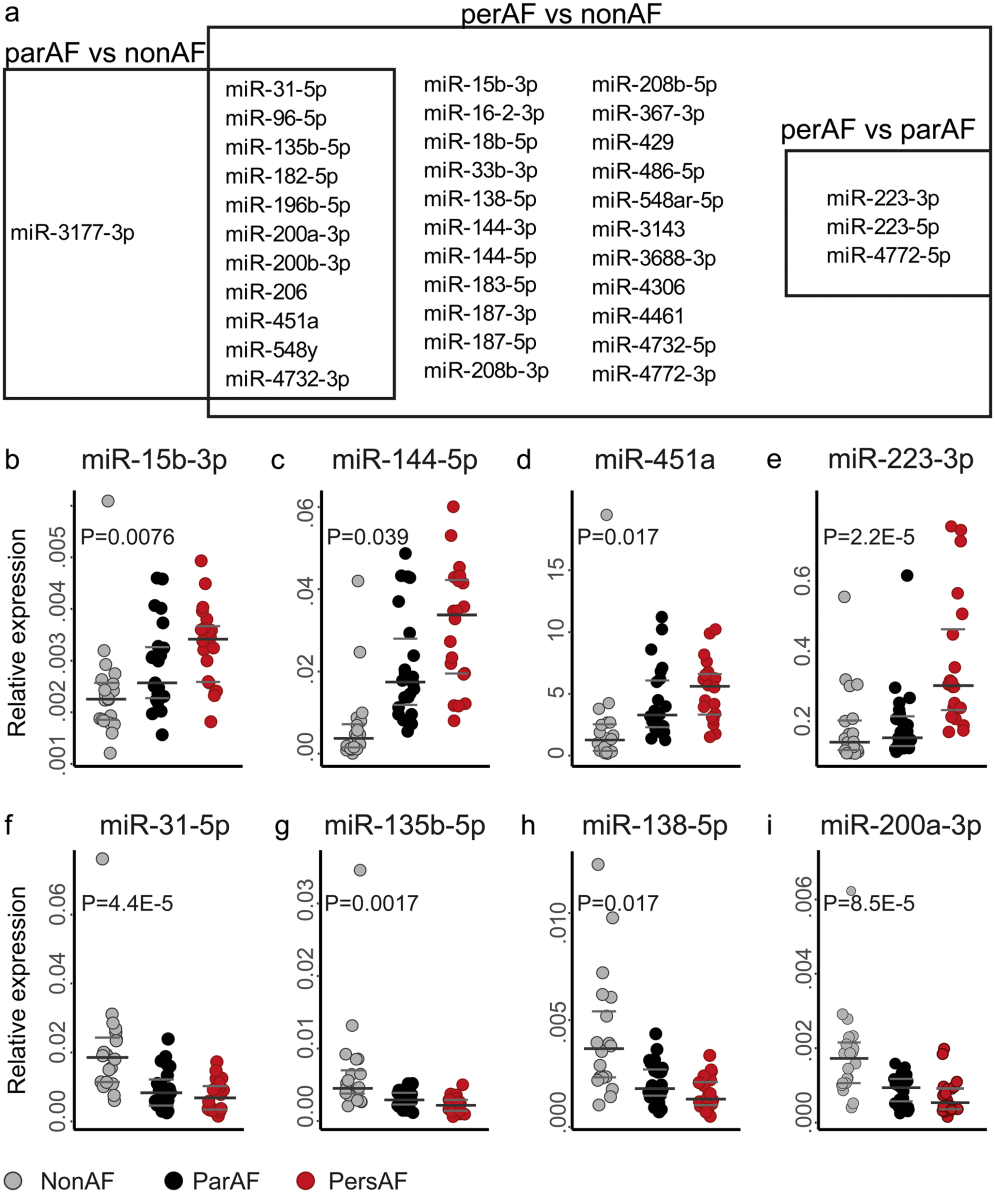
Top differentially expressed miRNAs in parAF and persAF. **a** Venndiagram showing in which comparison miRNAs were found to be differentially expressed (FDR<0.05 and |log2FC|> 1). **b**–**i** Representative results of miRNA qPCR validation. **b**–**e** upregulated miRNAs, **f**–**i** downregulated miRNAs. Note that one nonAF sample appears as an outlier. This is not one and the same sample in each figure. To demonstrate that the results were not significantly affected by these apparent outliers, Supplementary Fig. S3 shows the results with exclusion of these outliers. *P*-values represent the overall variation between the three groups. The miRNAs presented in the graph visually showed an overall ordinal increase or decrease in expression, for which a post-hoc analysis was considered irrelevant and too detailed. *P*-values were calculated with One-way-ANOVA or Kruskal-Walllis Test when appropriate. Other validated miRNAs can be found in supplementary data

There were 36 DE miRNAs with an absolute log2FC > 1 between either one of the three comparisons (Fig. 3a; Table 1). Most of these miRNAs were embedded in the comparison of persAF vs. nonAF (Fig. 3a), in line with the observed ordinal trend of miRNA expression from nonAF to parAF to persAF (Fig. 2b, c). Only few miRNAs showed a specific increase between parAF vs. nonAF (miR-206, miR-548y and miR-3177-3p) or persAF vs. parAF (miR-223-3p, miR-223-5p and miR-4772-5p) only. Twenty-two miRNAs out of 36 DE miRNAs with an absolute log2FC > 1 were selected for validation (AveExpr > 1) with qPCR (Table 1). Three miRNAs, those with the lowest expression levels, showed unreliable results, which precluded validation. The expression pattern of 18 out of 19 miRNAs (95%) was reproduced by qPCR (Fig. 3b–i, Supplementary Fig. S2, S3).

**Table 1:** Most differentially expressed tissue miRNAs associated with atrial fibrillation

	**AveExpr**	**LogFC persAF vs. nonAF**	**FDR persAF vs. nonAF**	**LogFC parAF vs. nonAF**	**FDR parAF vs. onAF**	**LogFC persAF vs. parAF**	**FDR persAF vs. parAF**
miR-486-5p	14.89	1.08	6.82E-03	0.65	0.175	0.43	0.455
miR-451a	12.24	2.18	3.76E-05	1.28	0.046	0.9	0.187
miR-144-5p	10.76	1.97	5.44E-04	1.1	0.118	0.86	0.275
miR-223-3p	9.58	1.74	3.76E-05	0.05	0.947	1.69	2.16E-04
miR-144-3p	8.93	2.29	3.76E-05	1.19	0.06	1.1	0.102
miR-16–2-3p	7.68	1.18	3.76E-05	0.62	0.066	0.56	0.123
miR-187-3p	7.61	1.21	1.95E-04	0.35	0.431	0.87	0.038
miR-208b-3p	7.4	1.27	0.023	0.46	0.53	0.81	0.263
miR-31-5p	6.67	−2.08	3.76E-05	−1.39	0.019	−0.68	0.316
miR-182-5p	6.62	2.08	3.76E-05	1.53	0.01	0.56	0.399
miR-15b-3p	5.39	1.15	6.23E-05	0.57	0.109	0.58	0.125
miR-200b-3p	4.03	−1.41	3.70E-04	−1.05	0.03	−0.36	0.541
miR-200a-3p	3.49	−1.54	1.05E-04	−1.24	0.011	−0.29	0.624
miR-196b-5p	2.73	1.89	3.76E-05	1.13	0.047	0.76	0.209
miR-18b-5p	2.57	1.03	5.26E-04	0.36	0.37	0.67	0.086
miR-223-5p	2.47	1.2	5.63E-04	−0.18	0.776	1.38	6.75E-04
miR-135b-5p	2.43	−2.41	3.76E-05	−1.54	0.019	−0.87	0.25
miR-138-5p	2.14	−1.08	1.59E-03	−0.49	0.235	−0.58	0.209
miR-548ar-5p	1.73	1.09	2.94E-03	0.71	0.118	0.38	0.492
miR-4306	1.63	1.71	4.83E-04	1.19	0.054	0.51	0.489
miR-206	1.54	1.15	1.05E-04	1.28	0	−0.13	0.803
miR-4732-3p	1.2	2.29	1.38E-04	1.7	0.026	0.59	0.513
miR-429	0.96	−1.45	1.33E-03	−1.05	0.054	−0.41	0.542
miR-3688-3p	−0.19	1.83	3.97E-05	1.11	0.054	0.71	0.252
miR-3143	−0.33	1.99	3.76E-05	0.95	0.13	1.05	0.099
miR-187-5p	−0.46	1.36	0.019	0.74	0.324	0.63	0.455
miR-4732-5p	−0.61	2.18	1.25E-04	1.42	0.054	0.75	0.367
miR-183-5p	−0.74	1.64	6.98E-03	0.78	0.34	0.86	0.316
miR-96-5p	−1.13	2.21	3.76E-05	1.38	0.047	0.82	0.266
miR-4772-3p	−1.32	1.71	4.03E-03	0.75	0.358	0.96	0.25
miR-548y	−1.57	−1.17	0.022	−1.37	0.023	0.2	0.83
miR-208b-5p	−1.73	1.79	9.83E-03	1.04	0.246	0.75	0.466
miR-4772-5p	−1.74	1.15	0.042	−0.41	0.613	1.57	0.02
miR-33b-3p	−2.13	−1.44	9.84E-03	−1.26	0.056	− 0.18	0.881
miR-3177-3p	−2.53	0.42	0.561	1.27	0.046	− 0.85	0.224
miR-367-3p	−2.92	−1.38	0.021	−0.65	0.369	− 0.73	0.358

MiRNAs are selected for FDR and log2FC and sorted by CPMs

*AveExpr* average log2CPM, *FDR* false discovery rate adjusted *P*-value, *LogFC* log2 fold changes

### MiRNA regulation of the atrial fibrillation mRNA signature

**Fig. 4:**
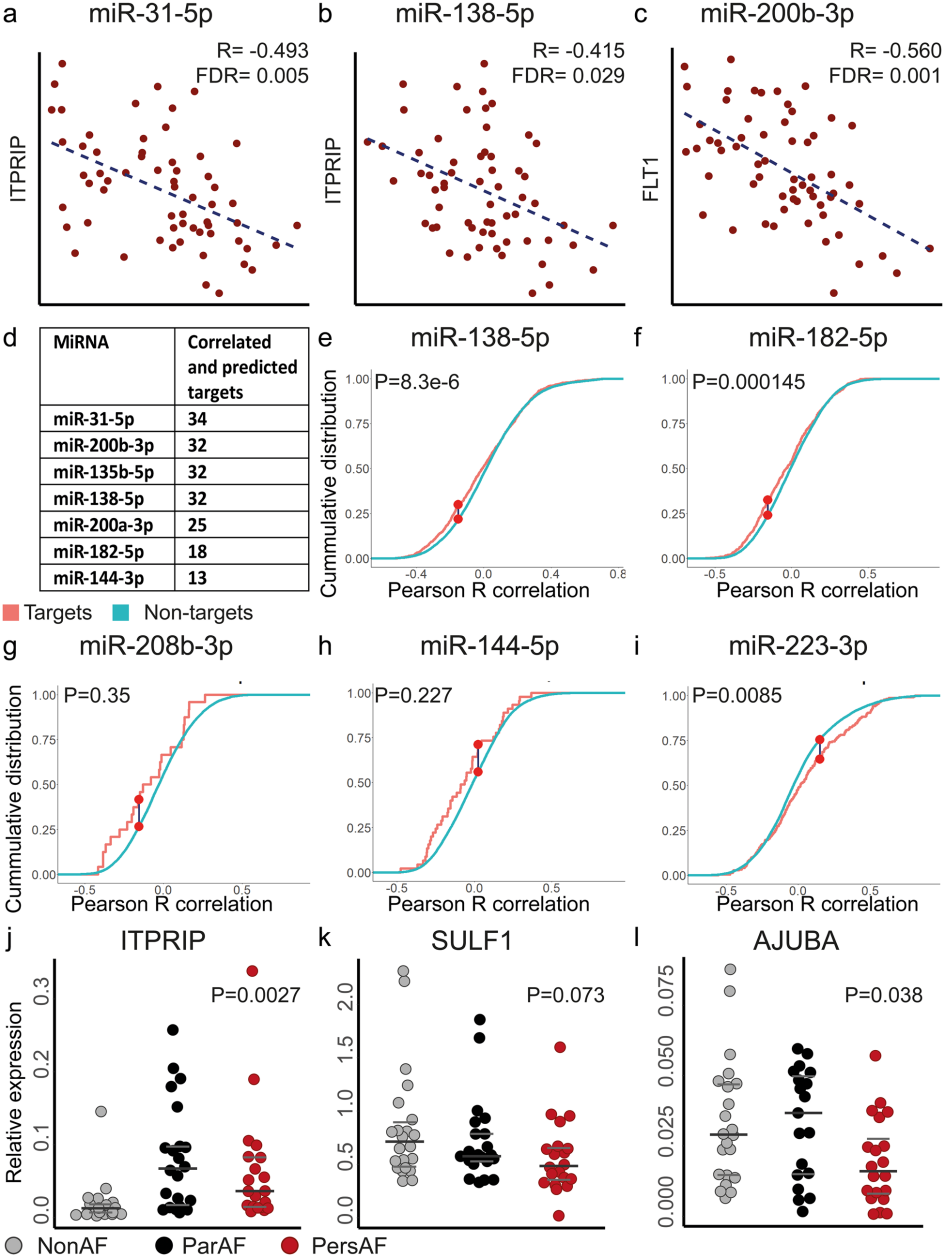
Top differentially expressed miRNAs that can regulate a multitude of target genes. **a**–**c** Correlation graphs show typical examples of miRNA–mRNA sequencing correlations. **d** Table showing differential expressed miRNAs with more than 10 predicted and correlated targets. **e**–**i** Cumulative distribution functions were plotted for predicted and non-targets (CPM > 3) of top differentially expressed miRNAs. *P*-values were calculated using a Kolmogorov–Smirnov test. **e** Typical example of a downregulated (miR-138-5p) and **f** upregulated (miR-182-5p) miRNA that show more negative correlations among predicted targets than among the non-targets. **g** MiR-208b-3p and **h** miR-144-5p typify miRNAs with fewer predicted targets. An effect of miRNAs on mRNA expression is suggested visually, but not significant. **i** MiR-223-3p exemplifies a miRNA with more positive correlations among its predicted targets. **j**–**l** qPCR quantification of predicted and correlated targets validated the upregulated expression of *ITPRIP* and downregulated expression of *AJUBA* and showed a tended decrease of *SULF1*. P-values represent the overall variation between the three groups and were calculated with one-way-ANOVA or Kruskal-Walllis test when appropriate

To assess the regulatory effects of DE miRNAs on mRNA expression, Pearson correlations were determined between the expression levels of 103 DE miRNAs (FDR < 0.05) and 17,324 mRNAs. Negative correlations (R < − 0.4 and FDR < 0.05) were cross-checked with miRNA–mRNA pairs from target prediction (Fig. 1). This resulted in 1017 miRNA–mRNA interactions found in the intersect of 48,904 predicted miRNA–mRNA pairs and 25,746 negatively correlated miRNA–mRNA pairs (examples Fig. 4a–c; full list in Supplementary Data file). The enrichment of predicted targets among the negatively correlated miRNA–mRNA pairs was 1.11 (*P* < 0.001), supporting a significant regulatory effect of miRNAs at the transcriptome level in AF. Conversely, predicted targets were not enriched among positively correlated miRNA–mRNA pairs (*R* > 0.4 and FDR < 0.05; enrichment 1.02; *P* = 0.16).

The top DE miRNAs validated by qPCR were assessed for individual miRNA effects at the transcriptional level. MiR-31-5p, miR-200b-3p, miR-135b-5p, miR-138-5p, miR-200a-3p, miR-182-5p and miR-144-3p had more than 10 predicted targets that were also negatively correlated with mRNA expression (Fig. 4d).

For the top DE miRNAs, we also determined whether predicted mRNA targets were more negatively correlated with the miRNA than non-predicted targets (Wang et al. 2019). MiR-31-5p, miR-200b-3p, miR-135b-5p, miR-138-5p, miR-200a-3p, miR-182-5p and miR-4306 were indeed more negatively correlated among predicted targets (CPM > 3) (Wang et al. 2019) than among non-predicted targets (Fig. 4e–f; Fig. S4 in Data Supplement) and miR-196b-5p trended towards having more negatively correlated targets (*P* = 0.054) (Fig. S4 in Data Supplement). Visualization of miR15b-3p, miR-144-5p and miR-208b-3p, which have fewer predicted targets, also suggested stronger negative correlations with predicted targets, but without statistical significance (Fig. 4g–h). Conversely, miR-223-3p, miR-486 and miR-548ar demonstrated (a trend towards) more positive correlations with their target genes than with non-predicted targets (Fig. 4i).

Note that the miRNAs, which show the most correlated and predicted targets, are mostly downregulated miRNAs (Fig. 4d). Correspondingly, downregulated miRNAs more often show a significant anti-correlation with multiple target genes than upregulated miRNAs (Fig. 4e–i; Supplementary Fig. S4).

Table 2 presents the top miRNA–mRNA interactions, selected for a negative Pearson *R* correlation (Fig. 4a–c), target prediction and DE of both the miRNA and the mRNA. Among the targets were three genes (*ITPRIP*, *MFAP3L* and *AJUBA*) that were predicted to be targeted by two DE miRNAs (miR-138-5p and miR-31-5p; miR15b-3p and miR18b-5p and miR-182-5p and miR18b-5p, respectively; Table 2). *ITPRIP, AJUBA* and *SULF1* were selected from Table 2 for validation of expression levels with qPCR as representative targets of various miRNAs (Fig. 4j–l).

**Table 2:** Top predicted and correlated miRNA–mRNA interactions in atrial fibrillation

**miRNA**	**Target**	**Pearson** ***R***	**FDR**	**Log2FC mRNA persAF vs. nonAF**	**FDR mRNA persAF vs. nonAF**	**Validated**	**Predicted**
miR-135b-5p	PPP1R12C	−0.482	0.007	0.625	0.000	No	Yes
miR-135b-5p	COL4A3	−0.463	0.011	0.558	0.000	Yes	Yes
miR-135b-5p	RNF152	−0.403	0.036	0.784	0.000	No	Yes
miR-138-5p	F2RL3	−0.457	0.012	2.462	0.000	No	Yes
miR-138-5p	ITPRIP	−0.415	0.029	1.128	0.000	No	Yes
miR-138-5p	HEYL	−0.476	0.008	1.119	0.000	No	Yes
miR-138-5p	KIAA0355	−0.465	0.011	0.516	0.000	No	Yes
miR-138-5p	CSPG4	−0.432	0.021	0.645	0.000	Yes	No
miR-138-5p	SLC62A2	−0.402	0.036	0.510	0.000	No	Yes
miR-144-3p	PEX5L	−0.421	0.026	−1.933	0.000	Yes	No
miR-144-3p	RBM43	−0.440	0.018	−0.649	0.000	No	Yes
miR-15b-3p	MFAP3L	−0.430	0.022	−0.587	0.000	No	Yes
miR-182-5p	EIF5	−0.427	0.023	−0.404	0.000	Yes	Yes
miR-182-5p	AJUBA	−0.408	0.032	− 0.631	0.000	Yes	Yes
miR-182-5p	MFAP3L	−0.421	0.026	−0.587	0.000	No	Yes
miR-18b-5p	PARD6B	−0.421	0.026	−1.199	0.000	Yes	Yes
miR-18b-5p	AJUBA	−0.452	0.014	−0.631	0.000	Yes	No
miR-18b-5p	MFAP3L	−0.531	0.002	−0.587	0.000	Yes	No
miR-200a-3p	SCHIP1	−0.415	0.029	0.543	0.001	No	Yes
miR-200a-3p	SOX17	−0.419	0.027	1.864	0.000	No	Yes
miR-200b-3p	FLT1	− 0.560	0.001	0.901	0.000	Yes	No
miR-200b-3p	PCDH19	− 0.410	0.031	0.665	0.000	No	Yes
miR-206	CA12	−0.475	0.008	−2.505	0.000	No	Yes
miR-206	WWC1	−0.507	0.004	−2.345	0.000	No	Yes
miR-206	SULF1	−0.452	0.014	−0.838	0.000	No	Yes
miR-206	LIX1	−0.400	0.037	−1.765	0.003	No	Yes
miR-206	MAL2	−0.516	0.003	−1.591	0.000	No	Yes
miR-31-5p	ITPRIP	−0.493	0.005	1.128	0.000	Yes	No
miR-31-5p	CASKIN2	−0.428	0.023	0.657	0.000	Yes	No
miR-31-5p	DACT3	−0.428	0.023	0.340	0.008	Yes	No
miR-4306	PRKAB2	−0.449	0.015	−0.545	0.000	No	Yes

Top miRNA–mRNA correlations out of 1017 negatively correlated and predicted pairs. Additional selection criteria were for the miRNAs to be DE at FDR < 0.05, |log2FC|> 1, CPM > 1 and mRNAs had to be DE at FDR < 0.05 and |log2FC|> 0.5 in any of the comparisons

### Biological processes associated with miRNA expression in atrial fibrillation

To investigate a potential function of the discovered miRNAs, gene set enrichment analysis of target genes was performed. We focused on the continuous processes in AF since the large majority of genes as well as miRNAs demonstrated an ordinal trend from nonAF to parAF to persAF.

Firstly, we determined which processes discovered by mRNA sequencing were regulated by miRNAs. Of the 270 biological processes discovered by mRNA sequencing, 32 processes were predicted to be regulated by a larger number of miRNAs than expected by chance (enrichment 2.4-times; Supplementary Data File and Supplementary Fig. S5) (Berg et al. 2021). Discovered processes were mostly upregulated in AF and included epithelial and endothelial cell differentiation and migration (e.g. *VEGFA*, *ROCK2*, *ETS1* and *MET*), ‘*regulation of RAC1 activity’* (e.g. *RAC1*, *ARHGAP6* and *KALRN*)*,* ‘*integrins in angiogenesis*’ (e.g. *VEGFA*, *AKT1* and *COL4A3*) and ‘*glycosaminoglycan biosynthesis*’ (e.g. *VCAN*, *B3GNT2*, *CHST11* and *CHSY1*) (Berg et al. 2021). Upregulated signalling pathways enriched in miRNA targets included *‘response to VEGF’* (e.g. *VEGFA*, *FOXC1*, *SPRY2* and *NOTCH1*) and processes involving interleukin signalling (e.g. *IL6R*, *JAK2*, *JUN* and *MAP3K8*) (Berg et al. 2021)*.*

**Fig. 5:**
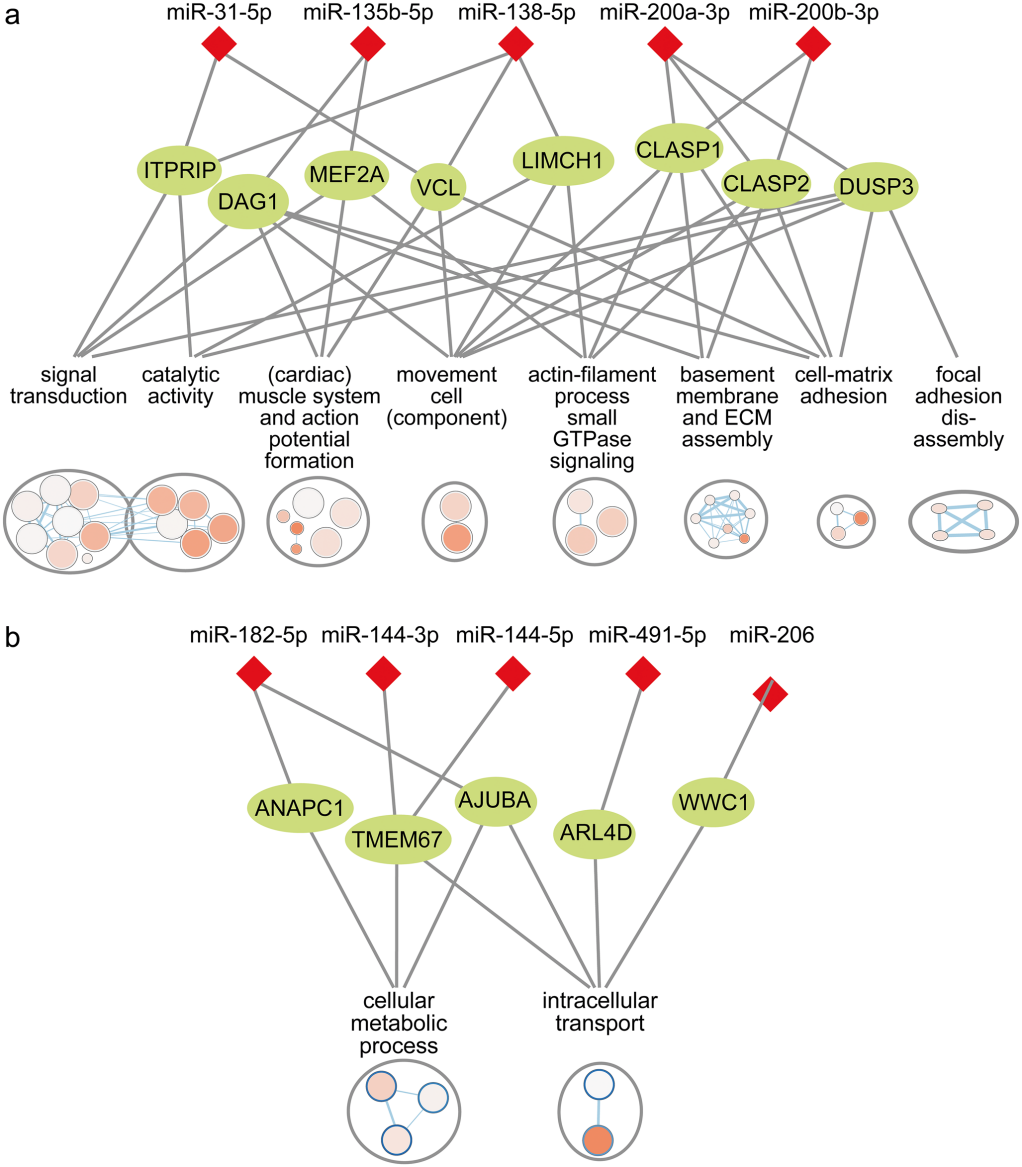
MiRNAs regulate biological processes involved in atrial fibrillation pathophysiology. **a** Biological processes enriched in correlated and predicted targets of the top 5 downregulated miRNAs. Only a small number of targets are shown. The figure illustrates that few miRNAs may target a multitude of genes and processes. **b** Biological processes enriched in correlated and predicted targets of upregulated miRNAs. Only a small number of miRNAs and targets are shown. The figure illustrates how multiple miRNAs may work synergistically to regulate one or few targets and processes

As a complementary approach, the negatively correlated and predicted targets of DE miRNAs were used for gene set enrichment. The top downregulated miRNAs (miR-31-5p, miR-200b-3p, miR-135b-5p, miR-138-5p and miR-200a-3p) had targets overrepresented in a variety of processes involved in or related to cell–matrix adhesion (Fig. 5a; Supplementary data file). Cell–matrix adhesion is a major upregulated process in the RNA sequencing analysis interconnecting endothelial and epithelial cell migration and angiogenesis with collagen and proteoglycan synthesis (Berg et al. 2021). Processes overrepresented in targets of downregulated miRNAs included “*cell-substrate junction organization”* (e.g. *DMD*, *VCL*, *DAG1* and *LIMCH1*), “*basement membrane assembly”* and “*positive regulation of extracellular matrix assembly”* (e.g. *NTN4*, *DAG1*, *CLASP1* and *CSPG4*)*.* Target genes were further overrepresented in the disassembly of cell–matrix interactions, e.g. “*Focal adhesion disassembly*” (*MAPRE2*, *IQSEQ* and *DUSP3*). Processes related to cell motility and reorganization of the actin cytoskeleton was enriched in upregulated miRNA targets including ‘*movement of cell or subcellular component*”, “*Ras protein signal transduction*” and “*actin filament-based process*” (e.g. *MEF2A*, *LIMCH1*, *CLASP1*,* KALRN* and *KIAA0355*). Consistently, cell motility and Ras signalling were discovered as upregulated processes in AF compared to nonAF by the RNA sequencing analysis. The top downregulated miRNAs further had targets enriched in signal transduction, upregulated catalytic activity and processes related to cardiac muscle development and action potential formation (e.g. ‘*cardiac muscle contraction’*, *SLC8A1*, *CAMK2D*, *CALM1* and *DMD*).

Targets of the top 17 upregulated miRNAs were enriched in the KEGG pathway “*Hippo signalling*” only (e.g. *AJUBA*, *BMPR1A*, *WWC1* and *PARD6B*). When using the correlated targets of all upregulated miRNAs (71 out of 103 DE miRNAs) for enrichment analysis, these were overrepresented in biological processes related to intracellular transport (e.g. *ARL4D*, *MPP5*, *CROT* and *UPF2*) and cellular metabolic process (e.g. *ANAPC1*, *AJUBA* and *TMEM67*) (Fig. 5b; Supplementary Data File).

Note that both strategies for gene set enrichment discovered more upregulated biological processes regulated by downregulated miRNAs, than downregulated biological processes regulated by upregulated miRNAs.

### MiRNA-135b-5p and miRNA-138-5p regulate genes involved in cell migration

**Fig. 6:**
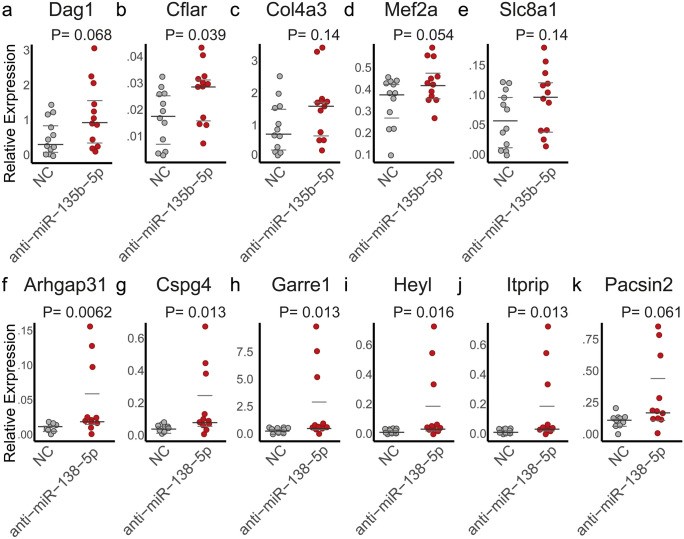
MiRNA inhibition upregulates multiple target genes. **a**–**e** Inhibition of miR-135b-5p demonstrated an increased expression of **b** *Cflar*, and a tended increase in the expression of **a** *Dag1* and **d** *Mef2a*. *P*-values were calculated using a *T*-test or Mann-Whitney U test. **f**–**k** Inhibition of miR-138-5p demonstrated an increased expression of **f**–**j** *Arhgap31*, *Cspg4*, *Garre1*, *Heyl*, and *Itprip*, and a tended increase in the expression of **k** *Pacsin2*. *P*-values were calculated using a non-parametric Mann-Whitney U test. Note that there are three outliers after miRNA inhibition. To demonstrate that the results were not substantially affected by these apparent outliers, Supplementary Fig. S6 shows the results with exclusion of these three outliers. NC, negative control

For the validation of a biological effect of the in silico discovered DE miRNAs on target gene expression and in to bring forward new hypotheses, we focused on two lesser known miRNAs in AF, miR-135b-5p and miR-138-5p (Xie et al. 2018). These miRNAs had targets involved in the processes discovered by RNA sequencing such as non-cardiomyocyte cell migration and differentiation, they were highly differentially expressed (decreased in AF), and had predicted targets that were significantly negatively correlated (Fig. 4d; Supplementary Fig. S3). Rat non-cardiomyocyte mesenchymal cells, including endothelial cells, epicardial cells and fibroblasts, were treated with a miR-135b-5p and miR-138-5p inhibitor. Target gene expression did not demonstrate high-fold changes or high significance after miRNA inhibition. However, all selected genes demonstrated an upward trend after miRNA inhibition, with the majority of target genes reaching statistical significance, consistent with miRNAs affecting multiple target genes simultaneously. Inhibition of miR-135b-5p (Fig. 6a–e) increased the expression of *Cflar* (Fig. 6b) and showed a tended increase of *Dag1* and *Mef2a* (Fig. 6a, d). Inhibition of miR-138-5p (Fig. 6f–k) increased the expression of target genes *Arhgap31*, *Cspg4*, *Garre1*, *Heyl* and *Itprip* (Fig. 6f–j) and demonstrated a tended increase of *Pacsin2* (Fig. 6f).

## Discussion

We here describe the relation between miRNAs and the mRNA signatures of AF. The combined miRNA and mRNA profiles from the same patients permit a direct assessment of whether mRNA profiles are actively regulated, as suggested by negatively correlated miRNA–mRNA expression. We discovered multiple miRNAs that have not previously been underscored as key regulators of the substrate of AF including miR-135b-5p, miR-200a-3p, miR-4306 and miR-182-5p, together with established miRNAs associated with AF pathophysiology such as miR-200b and miR-208b (van den Berg et al. 2017). The miRNA–mRNA pairs that showed a negative correlation were enriched in miRNA–mRNA pairs supported by target prediction. These data provide evidence for a broad regulatory function of miRNAs on a multitude of target genes in AF. Upregulated processes in AF discovered by the RNA sequencing analysis were enriched in miRNA targets, including epithelial and endothelial cell differentiation and glycosaminoglycan biosynthesis. Downregulated miRNAs were predicted to target and were negatively correlated with genes enriched in cell–matrix interactions, small GTPase signalling and actin-filament based process. These findings suggest that the structural remodelling processes discovered by RNA sequencing, characterized by endothelial to mesenchymal transition, endothelial cell proliferation and aberrant ECM deposits may for a large part be regulated by miRNAs. The current study provides limited functional data and further in vitro and in vivo studies are needed for more mechanistic insight. The discovered miRNAs and the suggested miRNA-regulated processes could therefore be of interest for further functional studies investigating miRNAs regulating AF pathophysiology as our data generate hypotheses on how miRNAs could function synergistically and affect multiple mRNAs under physiological conditions.

Several discovery studies have reported differentially expressed miRNAs in association with AF, but discrepancies in the directional change of miRNAs remain (van den Berg et al. 2017). The inconsistencies between studies regarding miRNAs in AF are likely the result of variations in study populations. For example, two other discovery studies reported a downregulation of miR-31 in AF (Nishi et al. 2013; Morishima et al. 2016), but Reilly et al. found miR-31 upregulated (Reilly et al. 2016). Reilley et al. in that same study, however, showed that the upregulation of miR-31 depended on the characteristics of patients included in the analysis (Reilly et al. 2016). We assessed miRNA expression in a large and clinically well-profiled cohort of patients without AF, with paroxysmal AF or persistent AF. This allowed the discovery of miRNAs that do not cluster based on comorbidities and overall show an ordinal change in miRNA and mRNA expression from nonAF to parAF to persAF (Berg et al. 2021). From a clinical perspective, the study is limited as the control group in the current study did not include healthy patients but mostly patients with coronary artery disease. Nevertheless, the ordinal trend of the discovered miRNAs suggests that the nonAF patients included form a valid control group and supports the discovery of the large majority of miRNAs which show an ordinal trend or significant difference between parAF and persAF. The continuous trend of miRNA and mRNA expression levels also suggests that the processes involving large numbers of genes and miRNAs are continuously increased or decreased in AF. Still, some singular miRNAs appeared specific for parAF vs nonAF or persAF vs parAF. For example, miR-223-3p and miR-223-5p appeared upregulated in persAF vs. parAF specifically, which may be of interest for future studies investigating AF progression.

The combined miRNA and mRNA profiles provide evidence for a broad regulatory effect of DE miRNAs on the mRNA signatures of AF by showing stronger negative correlations among predicted targets than among non-targets. Although a 1.11 enrichment of predicted miRNA–mRNA pairs among negatively correlated miRNA–mRNA pairs appears modest, this cannot be considered independently of the complex features of miRNA–mRNA regulation. Depending on the prediction algorithm and the used cut-off values, miRNAs are predicted to target a few or up to hundreds of RNAs in silico, but not all targets are repressed to the same extent (Bartel and Chen 2004; Seitz 2009).

Correspondingly, the in vitro inhibition of miR-135b-5p and miR-138-5p increased the expression levels of all predicted targets quantified, but only modestly. Due to the small fold changes and large variation, not all targets reached statistical significance. However, all targets showed the same directional changes and many of the predicted target genes were functionally related, suggesting that these miRNAs may not affect one target gene, but may exert their function through small effects on multiple targets.

Not only can miRNAs target multiple mRNAs, but various mRNAs were predicted to be targeted by more than one top DE miRNA (e.g. *AJUBA*, *ITPRIP* and *MFAP3L*), supporting the notion that miRNAs may function synergistically (Seitz 2009). Despite these complex features of miRNA–mRNA regulation, our data show a wide-ranging miRNA effect on the mRNA signatures underlying AF, and we highlight top DE miRNAs including downregulated miR-31-5p, miR-200b-3p, miR-135b-5p, miR-138-5p and miR-200a-3p and the upregulated miR-182-5p and miR-144-3p. These miRNAs were predicted to target a multitude of mRNAs and therefore appear key regulators of the AF mRNA signature that could become targets for future substrate targeted therapies.

Two of the top DE miRNAs, miR-223-3p and miR-548ar-5p, showed more positive correlations among predicted targets than among non-predicted targets. A possible explanation is the binding of the miRNAs to a sponge such as a long non-coding RNA or circular RNA, which can inhibit miRNA function without miRNA degradation (Ebert et al. 2007; Seitz 2009; Bang et al. 2014). A more theoretical explanation is miRNA secretion into the plasma at the expense of intracellular atrial miRNAs (Creemers et al. 2012) or alternatively an independent upregulation in plasma levels is surpassing tissue downregulation. It is not unlikely that, despite washing, some blood remained in the bulk tissues. MiR-223 is enriched in platelets, leukocytes and exosomes, which could fit with increased plasma levels (Dickinson et al. 2013; Poon et al. 2017), but does not explain a depletion in atrial tissue.

Gene set enrichment analysis of miRNA targets strongly suggests miRNA modulation of structural remodelling in AF. The RNA sequencing analysis earlier discovered that structural remodelling encompassed EMT and endothelial cell proliferation and migration which was associated with increased interstitial (myo)fibroblast-like cell numbers and a disordered and excessive ECM (Berg et al. 2021). The discovered DE miRNAs appeared to interact with targets involved in many of the same processes including epithelial and endothelial cell migration, cell–matrix interactions and glycosaminoglycan synthesis. The top downregulated miRNAs miR-31-5p, miR-200b-3p, miR-135b-5p, miR-138-5p and miR-200a-3p in particular were negatively correlated with predicted target mRNAs involved in cell differentiation and migration (Lamouille et al. 2014)*.* MiR-135b-5p in vitro inhibition demonstrated an upregulation of the transcription factor *Mef2a*, which is associated with endothelial dysfunction and differentiation of vascular smooth muscle cells (Zhao et al. 2012). The inhibition of miR-138-5p resulted in an upregulated *Garre1*, *Arhgap31* and *Pacsin2*, which affect cytoskeletal dynamics through Rho GTPases and *RAC1* (Southgate et al. 2011; de Kreuk et al. 2011; Bagci et al. 2020). MiR-138-5p inhibition further resulted in an upregulated *Heyl,* an important notch-effector gene and a regulator of EMT (Fischer et al. 2007). Furthermore, miRNAs were predicted to upregulate processes such as basement membrane assembly and glycosaminoglycan synthesis, suggested miRNA regulation of ECM composition. For example, we found miR-135b-5p targeting basement membrane type IV collagen and found miR-138-5p targeting CSPG4. Moreover, miR-206 was associated with the downregulation of *SULF1*, which regulates the post-translational modification of heparin sulphate proteoglycans and can thereby affect VEGF and angiogenic signalling (Korf-Klingebiel et al. 2019).

The suggested biological implications of DE miRNAs in the regulation of EMT and endothelial cell proliferation are corroborated by previous reports from miRNA functional studies. For example, miR-182-5p and miR-18b-5p were found upregulated and predicted to target a downregulated *AJUBA. AJUBA* is a corepressor of *SNAI1*, a key upregulated transcription factor of EMT and endothelial cell proliferation and differentiation (Ayyanathan et al. 2007). Moreover, the miRNA-200 family (DE were miR-200a-3p, miR-200b-3p and miR-429) has previously been shown to repress translation of the EMT transcription factor *ZEB1* mRNAs (that was upregulated) (Gregory et al. 2008). MiRNA-200 has also been found enriched in the cardiac endothelium where it regulates endothelial cell proliferation in angiogenesis (Magenta et al. 2017) and miR-200b directly targets *ETS1*, a pro-angiogenic transcription factor (Magenta et al. 2017). MiR-31 has been shown to target key genes of cell migration such as *RHOA* and *ITGA5* and attenuated breast cancer cell invasion (Valastyan et al. 2010).

In this study, the downregulated miRNAs miR-31-5p, miR-200b-3p, miR-135b-5p, miR-138-5p and miR-200a-3p had multiple anti-correlated targets and were shown to have a regulatory role in modifying the structural remodelling processes. The biological implications of upregulated miRNAs were less clearly defined. Our analysis was based on miRNA–mRNA interactions that were selected by target prediction and statistically anti-correlating expression levels. The dominancy of downregulated miRNAs could be a statistical effect, driving the discovery of relevant miRNA–mRNA interactions towards the discovery of target genes and processes that had significant variance in opposite direction. A biological explanation for the profound role of downregulated miRNAs in this study is that the downregulation of a miRNAs may be particularly effective to regulate large shifts in gene expression as seen in EMT and in cell differentiation and migration. The efficacy of those processes benefits from stable gene targets with low rates of turnover (Bartel and Chen 2004; Lamouille et al. 2014).

It is important to point out that the current study is associative in nature. For a full understanding of the underlying mechanisms of miRNA regulation, it is essential that the presented results of a single or possibly a panel of miRNAs are validated in future functional models including specific human atrial cell cultures, luciferase binding assays and in vivo studies. Here, our objective was to exploit the properties of human atrial tissues to the fullest and be hypothesis-generating. Human tissues offer the opportunity to employ an unbiased strategy to investigate multiple miRNAs and to investigate the way these miRNAs could function synergistically by targeting many genes simultaneously. As a result, the presented data do not provide details on miRNA effect size nor can we determine the relevance of individual miRNAs. Instead, the presented data provide insight into the full complexity of miRNA regulation under physiological conditions. Furthermore, we determined miRNA effects at the transcriptome level, through degradation of the target mRNA, only. MiRNAs can also repress translation with regulatory effects seen at the protein level. Our study also did not validate the direct binding of miRNAs to target mRNAs and it is possible that some of the described effects are indirect regulatory effects. Luciferase assays may be useful in future functional studies investigating regulatory pathways and few miRNAs in more detail. Finally, we used bulk tissues for miRNA and transcriptome sequencing. Many of our discoveries were suggestive of a function of the miRNA in epicardial cells, endocardial cells or fibroblasts, but cell type–specific miRNA regulation needs to be taken into account in future studies. Because of these limitations, we could not provide a completed miRNA–mRNA regulatory network, and it is possible that some functional miRNAs have not been identified. Altogether, our study shows a regulatory effect of multiple miRNAs on gene expression and provides concrete miRNA–mRNA interactions involved in structural AF substrate changes.

Overall, our mostly descriptive data provides evidence for a broad effect of DE miRNAs on the mRNA signatures of AF patients. Downregulated miRNAs miR-31-5p, miR-200b-3p, miR-135b-5p, miR-138-5p and miR-200a-3p were associated with upregulated EMT and endothelial cell proliferation and migration. These processes are implicated in extensive (myo)fibroblast activation and may underlie atrial fibrotic remodelling in the setting of AF. Several of these miRNAs with evidence for a regulatory function in AF structural remodelling are of interest for further functional study and are potential targets for future therapies.

### Supplementary Information

Below is the link to the electronic supplementary material.

Supplementary file1 (XLSX 340 KB)

Supplementary file2 (DOC 3069 KB)

## Data Availability

The transcriptome sequencing data and miRNA sequencing data that supported this study can be found in the European Genome-Phenome Archive (EGA), under EGAS00001005295 and EGAS00001005296, respectively. All data will be made available upon reasonable request to the corresponding author.
