# Supplementary material for: MicroRNAs in atrial fibrillation target genes in structural remodelling
Source: Cell Tissue Res. 2023 Oct 14;394(3):497–514. doi: 10.1007/s00441-023-03823-0 (PMC10697892; doi:10.1007/s00441-023-03823-0)
Supplement: Supplementary file 2 — Supplementary file2 (DOC 3069 KB) [file 441_2023_3823_MOESM2_ESM.doc]

**SUPPLEMENTARY MATERIALS**

# MicroRNAs in atrial fibrillation target genes in structural remodelling

Nicoline W.E. van den Berg, MD*, Makiri Kawasaki, PhD*, Fransisca A. Nariswari, MSc*, Benedetta Fabrizi, MSc*, Jolien Neefs, MD, PhD*, Ingeborg van der Made, MSc*, Robin Wesselink, MSc*, Wim Jan P. van Boven, MD, PhD*, Antoine H.G. Driessen, MD, PhD*, Aldo Jongejan, PhD‡, Joris R. de Groot, MD, PhD*

* Amsterdam UMC, University of Amsterdam, Heart Center; department of Clinical and Experimental Cardiology, Amsterdam Cardiovascular Sciences, Meibergdreef 9, Amsterdam, The Netherlands

‡ Amsterdam UMC, University of Amsterdam, Bioinformatics Laboratory, Clinical Epidemiology, Biostatistics and Bioinformatics, Meibergdreef 9, Amsterdam, The Netherlands

**Supplementary Materials and Methods**

**Library preparation for transcriptome sequencing**

Transcriptome libraries were prepared from 250ng of total RNA after rRNA depletion with QIAseq FastSelect (Qiagen). Strand-specific sequencing libraries were prepared using rRNA depletion with the NEBNext rRNA Depletion kit. cDNA synthesis was performed with the NEBNext RNA First Strand Synthesis, NEBNext Ultra Directional RNA Second Strand Synthesis Modules, NEBNext Directional RNA Library Prep Kit for Illumina and the NEBNext Ultra II DNA Library Prep Kit for Illumina (New England BioLabs). Library quantification was performed with the Kapa Illumina GA Revised Primers-SYBR Fast Universal kit (Kapa Biosystems). Average size fragment were determined (LabChip GX, PerkinElmer).

**Library preparation for miRNA sequencing**

MiRNA libraries were prepared from 75ng of total RNA with the TailorMix miRNA Sample Prep Kit (Seqmatic). Total RNA was used for 3’ Adapter Ligation and bound to TailorMag Purification Beads. 3’ adapter ligated TailorMag Purifaction Beads were ligated with 5’ Adapters and used for cDNA synthesis and PCR amplification. cDNA samples were size selected with gel electrophoresis (8% PAGE gel). Size selected miRNA libraries were extracted from the 140bp band.

**Transcriptome sequencing data analysis**

Reads were mapped towards the human reference genome (GRCh38v94) using HiSAT2 v2.1.0 with default settings. Uniquely mapped reads were counted with HTSeq v0.11. Genes were filtered out that showed a minimum expression of two counts per million in five or more samples. Normalization of read counts was performed using the Trimmed mean of M-values (TMM) method (R package *EdgeR*).

**MiRNA sequencing data analysis**

After adapter trimming, reads were collapsed and mapped to the reference genome (Ensembl 75) using bowtie. MiRNAs that did not show a minimum expression of two counts per million in 15 or more samples were filtered out. Mapped reads were annotated to possible contaminants (tRNA fragements, rRNA, sn(o)RNA, piRNA) and subsequently to mature miRNAs using genome annotation data from Ensembl 75, UCSC and miRBase v21. We used raw, mature miRNA expression data (sum of all isomiR reads from the canonical mature miRNA locus) for differential expression analysis. Normalization of read counts was performed using the Trimmed mean of M-values (TMM) method (R package *EdgeR*).

**Construction of miRNA-mRNA target gene database**

We downloaded four miRNA-mRNA prediction databases (miRDB, TargetScan, DIANA-microT, RNA22) and two databases containing validated targets (miRTarBase, Tarbase) between August 20 and August 25, 2019. None of the prediction algorithms or databases was more than 10 years old. Details of the databases are described in Supplementary Table 1.

Databases were merged after converting miRNA and gene labels to miRBase v22 and Ensembl gene ID (Ensembl 75, UCSC)(Biomart). To come to an integrated score for the combined prediction databases, we first defined a prediction threshold for each of the databases (Supplementary Table S1). MiRNA-mRNA pairs that were predicted by multiple databases were considered the strongest, though more weight was given to validated target databases. A miRNA-mRNA interaction was defined as being predicted if it occurred in at least two out of four prediction databases or in either one of the validated databases and at least one prediction database.

**Enrichment of miRNA-mRNA interactions using permutation analyses**

We hypothesized that a significant proportion of the miRNA-mRNA negative correlations results in miRNA degradation of the mRNA, thus affecting mRNA expression signatures. To test this hypothesis, we assessed whether miRNA-mRNA pairs that exceeded the prediction score threshold criteria, were more common among the negatively correlated genes than would be expected by chance.

We limited our analysis to 103 miRNAs that were DE and combined these with all 17324 detected protein coding genes. We defined *x* as the set of miRNA-mRNA pairs that exceeded the target prediction score threshold. We defined *y* as the subset of miRNA-mRNA pairs that were negatively correlated (R< -0.4 and FDR< 0.05). We defined *n* as the number of miRNA-mRNA pairs present in both *x* and *y.* The null distribution of *n* was estimated using a permutation procedure (x1000). For each iteration step *j*, the miRNAs and mRNAs in *x* were randomly shuffled to make new pairs which were used to determine *n_j_*. Enrichment was determined by *n/mean of (n_1_ t/m n_1000_)*. The significance of the enrichment was determined with a one-sided permutation test in its null distribution.

Permutation testing was also used to determine enrichment of top DE miRNAs (FDR< 0.05; |log2FC| >1) among the miRNAs predicted to target genes in upregulated or downregulated biological processes. We defined *a* as the set of all miRNA-mRNA pairs (857 miRNA vs 17324 mRNA) that exceeded the target prediction score threshold. We previously performed gene set enrichment analysis using ranked gene lists of DE mRNAs (persAF vs. nonAF) discovering 270 upregulated or downregulated gene sets (GS) sized 30-250 genes at FDR<0.05. For each of the discovered gene sets, we extracted *GS_i_*, which we defined as the genes per gene set (GS) *i*. We extracted all genes per gene set (opposed to leading edge or DE mRNAs) in order to allow for the discovery of both up and downregulated miRNAs with moderate (i.e. non-significant) gene expression effects. We defined *b_i_* as the subset of *a*, in which the miRNA-mRNA pairs included a top DE miRNA as well as an mRNA from *GS_i_.* For each *GS_i_* the null distribution of *b_i_* was determined using a permutation procedure (x1000)^1^. For each iteration step, the miRNAs in *a* were randomly shuffled to make new pairs which were used to determine *GS_i,j_*. The significance of the enrichment was determined with a one-sided permutation test in its null distribution.

**Neonatal rat ventricular non-cardiomyocyte isolation**

All animal experiments were approved by the local Animal Experiments Committee (Academic Medical Center, University of Amsterdam) and carried out in compliance with the Guide for the Care and Use of Laboratory Animals and in accordance with national and institutional guidelines. Neonatal rat ventricular non-cardiomyocytes were isolated from 1-to-2-day-old Wistar rats (Janvier labs). Pups were anesthetized by isoflurane and hearts were excised after decapitation. Ventricles were cut into pieces and dissociated with trypsin (1 mg/mL;Sigma), and collagenase type 2 (1 mg/mL;Worthington). Cells were collected and resuspended in culture medium (M199 medium, Gibco) supplemented with 10% heat inactivated fetal bovine serum (FBS, Gibco), 1% HEPES (Gibco), 5000 U/L penicillin-G (Sigma), 2 mg/L vitamin B12 (Sigma-Aldrich), 3.5 g/L glucose, 1% non-essential amino acids (Gibco), 1% L-glutamine (Gibco). The suspensions were seeded, washed multiple times to remove cardiomyocyte contamination and cultured for 48h before RNA extraction.

**Supplementary Tables**

**Supplementary** Table S1. Patient characteristics of miRNA sequencing

|  | **All patients**  **n=62*** | **NonAF**  **n=20** | **ParAF**  **n=22** | **PersAF**  **n=20** |
| --- | --- | --- | --- | --- |
| Sex, female, n(%) | 14 (22.6) | 5 (25) | 5 (22.7) | 4 (20.0) |
| Age, years (±SD) | 62.6 ±9.1 | 65.7 ±8.0 | 60.0 ±8.3 | 62.2 ±10.3 |
| BMI, kg/m^2^ (±SD) | 27.2 ±3.4 | 28.6 ±3.1 | 26.1 ±3.9 | 26.9 ±2.8 |
| Vascular heart disease, n(%) | 20 (32.3) | 15 (75) | 1 (4.6) | 4 (20) |
| Myocardial infarction, n(%) | 8 (13) | 5 (25) | 1 (4.6) | 2 (10.0) |
| PCI, n(%) | 4 (6.5) | 2 (10) | 1 (4.6) | 1 (5.0) |
| CHA_2_DS_2_-VASc [IQs]† | 2.0 [1.0-3.0] | 3.0 [2.0-4.0] | 1 [0.25-1.75] | 1 [0.25-1.75] |
| CHA_2_DS_2_-VASc ≥4 | 9 (14.5) | 7 (35) | 1 (4.5) | 1 (5.0) |
| Vascular disease, n(%)‡ | 20 (32.3) | 15 (75) | 1 (4.6) | 4 (20) |
| Hypertension, n(%) | 33 (53.2) | 15 (75) | 8 (36.4) | 10 (50.0) |
| Diabetes Mellitus, n(%) | 6 (9.7) | 5 (25) | 0 | 1 (5.0) |
| Congestive heart failure, n(%) | 0 | 0 | 0 | 0 |
| Stroke/TIA/embolus, n(%) | 10 (16.1) | 6 (30) | 2 (9.1) | 2 (10.0) |
|  |  |  |  |  |
| **Echocardiography** |  |  |  |  |
| LAVI, ml/m^2^ (±SD) | 36.9 ±12.6 | 31.4 ±9 | 36.1 ±11 | 42.4 ±14 |
| LVEF, % (±SD) | 51.1 ±16.3 | 48.4 ±11 | 58.6 ±10 | 54.0 ±10 |
|  |  |  |  |  |
| **Medication** |  |  |  |  |
| Antiplatelet, n(%) | 18 (29) | 17 (85) | 1 (4.6) | 0 |
| Anticoagulation, n(%) | 42 (67.7) | 0 | 22 (100) | 20 (100) |
| ACE inhibitors, n(%) | 20 (32.3) | 9 (45) | 6 (27.3) | 5 (25) |
| Angiotensin receptor blockers, n(%) | 15 (24.2) | 5 (25) | 4 (18.2) | 6 (30) |
| Class IA AAD | 5 (8.1) | 0 | 4 (18.2) | 1 (5.0) |
| Class IC AAD | 17 (27.4) | 0 | 9 (40.9) | 8 (40.0) |
| Class II AAD | 31 (50) | 12 (60) | 9 (40.9) | 10 (50.0) |
| Class III AAD | 13 (21) | 1 (5.0) | 8 (36.4) | 4 (20.0) |
| Class IV AAD | 5 (8.1) | 0 | 2 (9.1) | 3 (15.0) |
| Digoxine, n(%) | 8 (13) | 0 | 3 (13.6) | 5 (25.0) |

*The transcriptome sequencing included 64 patients. Two patients were later added to the transcriptome analysis to allow subgroup analysis outside the scope of this study.

†A CHA_2_DS_2_-VASc ≥2 was an inclusion criteria for the PREDICT-AF study, in which left atrial appendages of nonAF patients were collected.

‡All patients with vascular disease (as in CHA_2_DS_2_-VASc) had vascular heart disease.

Abbreviations: AAD, a ntiarrhythmic drugs; AF, atrial fibrillation; BMI, body mass index; IQ, interquartiles; LAVI, left atrial volume index; LVEF, left ventricular ejection fraction; PCI, percutaneous coronary intervention; TIA, transient ischemic attack

**Supplementary Table S2. Characteristics of MiRNA-mRNA interaction databases**

| **Database** | **Prediction criteria** | **Predicted or validated**  **miRNA-mRNA pairs** | **Pairs for 103 DE miRNAs** | **Ref.** |
| --- | --- | --- | --- | --- |
| **miRDB v6.0** | mirDB score ≥75 | 211188 | 52141 | 2 |
| **TargetScan v7.2** | Total context score ≥-0.30 | 282409 | 57829 | 3 |
| **DIANA-microT v5.0** | Diana score ≥0.85 | 254879 | 60166 | 4 |
| **RNA22 v2.0** | RNA22 score ≥0.02  Free energy ≤ -18 | 2726181 | 497558 | 5 |
| **MiRTarBase v7.0** | *NA* | 153652 | 41647 | 6 |
| **DIANA-TarBase v7.0** | *NA* | 256371 | 75527 | 7 |

**Supplementary Table S3. MiRNA primers used for real-time PCR quantification**

| **MicroRNAs** | **Accession Code** | **Primer sequence 5’-3’** |
| --- | --- | --- |
| **miR-135b-5p** | MIMAT0000758 | TATGGCTTTTCATTCCTATGTG |
| **miR-138-5p** | MIMAT0000430 | AGCTGGTGTTGTGAATCAGGC |
| **miR-144-3p** | MIMAT0000436 | TACAGTATAGATGATGTACT |
| **miR-144-5p** | MIMAT0004600 | GGAUAUCAUCAUAUACUGUAAG |
| **miR-15b-3p** | MIMAT0004586 | CGAATCATTATTTGCTGCTCT |
| **miR-16-2-3p** | MIMAT0004518 | CCAATATTACTGTGCTGCTTTA |
| **miR-182-5p** | MIMAT0000259 | TTTGGCAATGGTAGAACTCACAC |
| **miR-187-3p** | MIMAT0000262 | TCGTGTCTTGTGTTGCAGCC |
| **miR-18b-5p** | MIMAT0001412 | TAAGGTGCATCTAGTGCAGTTA |
| **miR-196b-5p** | MIMAT0001080 | TAGGTAGTTTCCTGTTGTTGG |
| **miR-200a-3p** | MIMAT0000682 | TAACACTGTCTGGTAACGATG |
| **miR-223-3p** | MIMAT0000280 | TGTCAGTTTGTCAAATACCCC |
| **miR-223-5p** | MIMAT0004570 | CGTGTATTTGACAAGCTGAGT |
| **miR-200b-3p** | MIMAT0000318 | TAATACTGCCTGGTAATGATGA |
| **miR-208b-3p** | MIMAT0004960 | ATAAGACGAACAAAAGGTTTGT |
| **miR-31-5p** | MIMAT0000089 | AGGCAAGATGCTGGCATAGCT |
| **miR-4306** | MI0015836 | TGGAGAGAAAGGCAGT |
| **miR-451a** | MI0001729 | AAACCGTTACCATTACTGAGTT |
| **miR-486-5p** | MIMAT0002177 | TCCTGTACTGAGCTGCCCCGA |
| **miR-548ar-5p** | MIMAT0022265 | AAAAGTAATTGCAGTTTTTGC |
| **miR-191-5p** | MIMAT0000440 | CAACGGAATCCCAAAAGCAGCTG |
| **miR-27a-3p** | MIMAT0000084 | TTCACAGTGGCTAAGTTCCG |
| **miR-let7a-5p** | MIMAT0000062 | TGAGGTAGTAGGTTGTATAG |

**Supplementary Table S4. Gene primers used for real-time PCR quantification**

| **mRNA target gene** | **Forward Primer sequence 5’-3’** | **Primer sequence 5’-3’** |
| --- | --- | --- |
| *GUSB* | GCGTCCCACCTAGAATCTGC | ATCCACATACGGAGCCCCC |
| *HPRT1* | TGACACTGGCAAAACAATGCA | GGTCCTTTTCACCAGCAAGCT |
| *PGK1* | CGACCCTTCCTGGCCATC | GTGCCAATCTCCATGTTGTTGAG |
| *AJUBA* | TGGCTCTGTGTACTGTGAGG | CCCATTGCTTGTAGGATCTTCTC |
| *ITPRIP* | TCTGGACAAAGGACAAGGCTG | CAGCTCCAAACAAACTGCTGG |
| *SULF1* | TCACCCATTCAAGGAGGCTG | CCAGTGGTTGTTGTCATGCG |
| *Hprt** | CAGTACAGCCCCAAAATGGT | TCCACTTTCGCTGATGACAC |
| *Dag1* | GACTTCCAGGAGGCTGTTCC | CCTGCTGCAGACACCTTGAT |
| *Cspg4* | CTCCAGTTCTCCACATCGCA | CAGGGCAAGTCTGACCTGTAG |
| *Heyl* | AAGAAGCGCAGAGGGATCAT | TTCTCAAAGGCAGTGGGGAC |
| *Col4a3* | CCTGAAGGAACACAGCCACT | CTGCCCAGAGTACCAAGGTC |
| *Arhgap31* | CTCGGGACAGGATGTTCCAT | ATCTGAGCCGAACTCTTGCC |
| *Pacsin2* | GCCACAGTTTGAGGAGTGGT | GGGACACTAAGGTTGCTGCT |
| *Cflar* | TGCTTTCGGTTTCTAGGCGT | GATCGAACGAGACACGGTCC |
| *Slc8a1* | GAACCTCAGTGCCAGACACA | CGTTGCTTCCGGTGACATTG |
| *Mef2a* | TGTTTCAGTACGCCAGCACT | CCTTCTTGTTCAGAGCCTCCA |
| *Garre1* | ACGGTCCCTGTGCAAATAGG | GCTGGAACATGGTATGCGTC |
| *Itprip* | TCCATCACTTCTTCGTGGGC | TGTGACGGTAAAGAGTCCGC |

**Supplementary Figures**

**Supplementary Figure S1. Multidimensional scaling identified one nonAF outlier.**
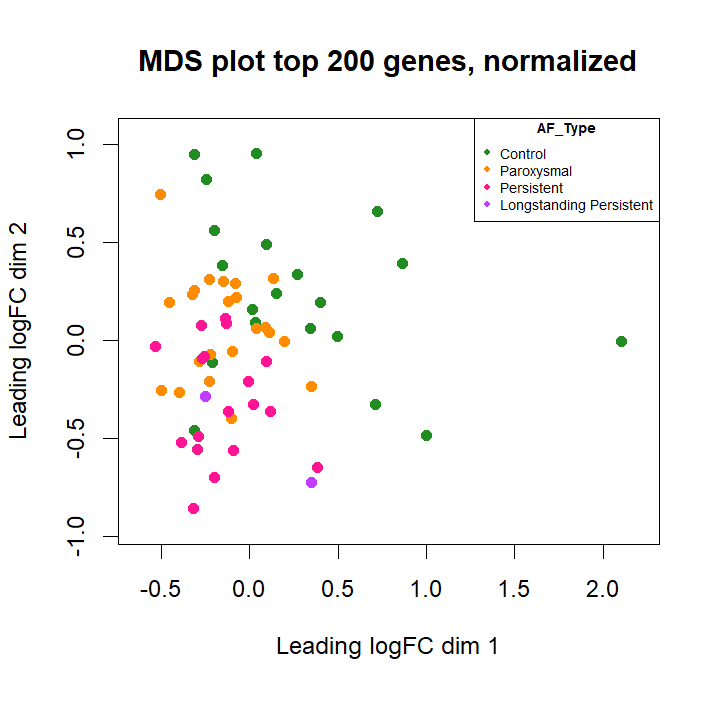


The nonAF outlier (right side) was removed from further analyses.

**Supplementary Figure S2. miRNA expression validation with qPCR (continued)**

**
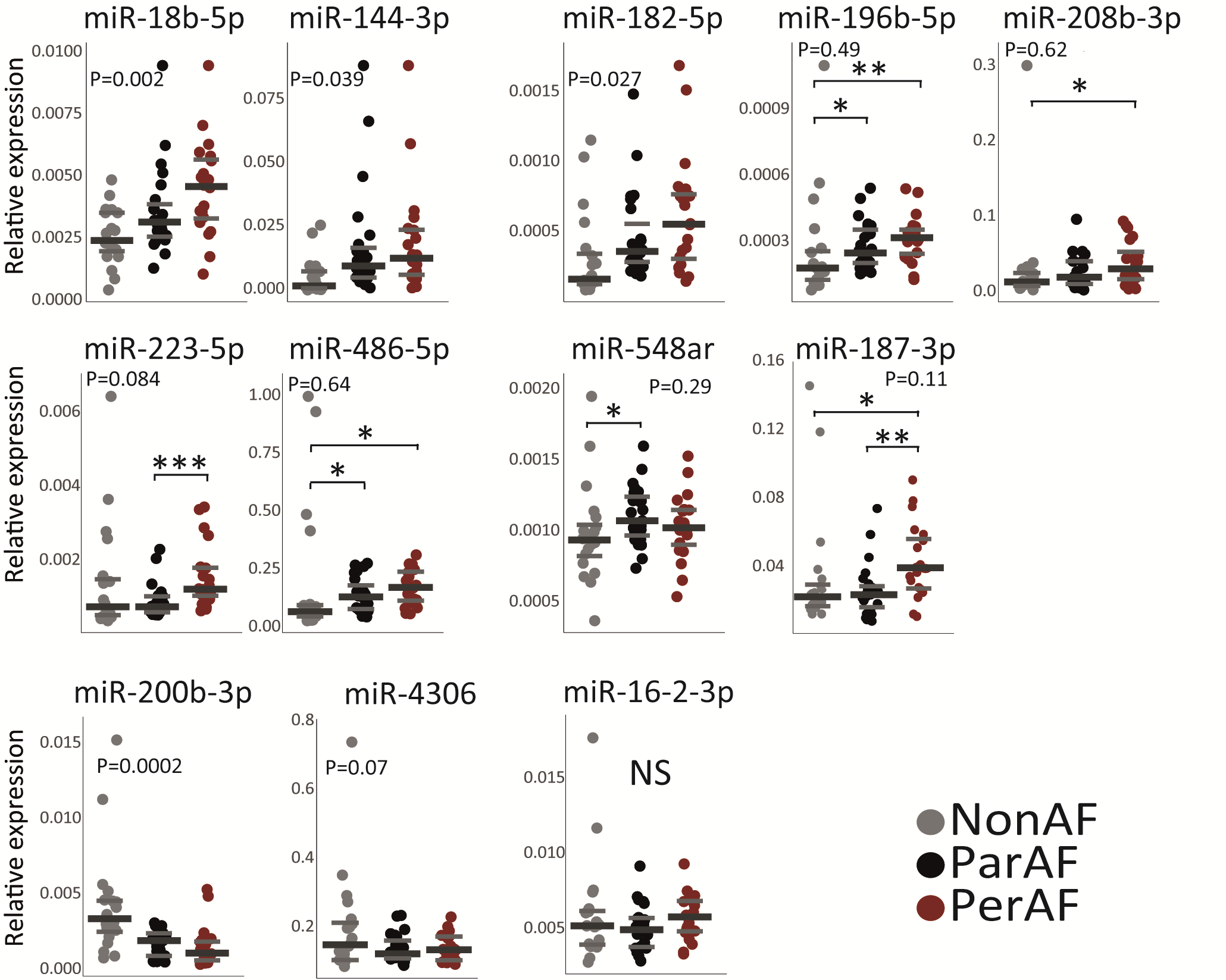
**

**Supplementary Figure S3. miRNA expression can be validated also after exclusion of outliers.**

**
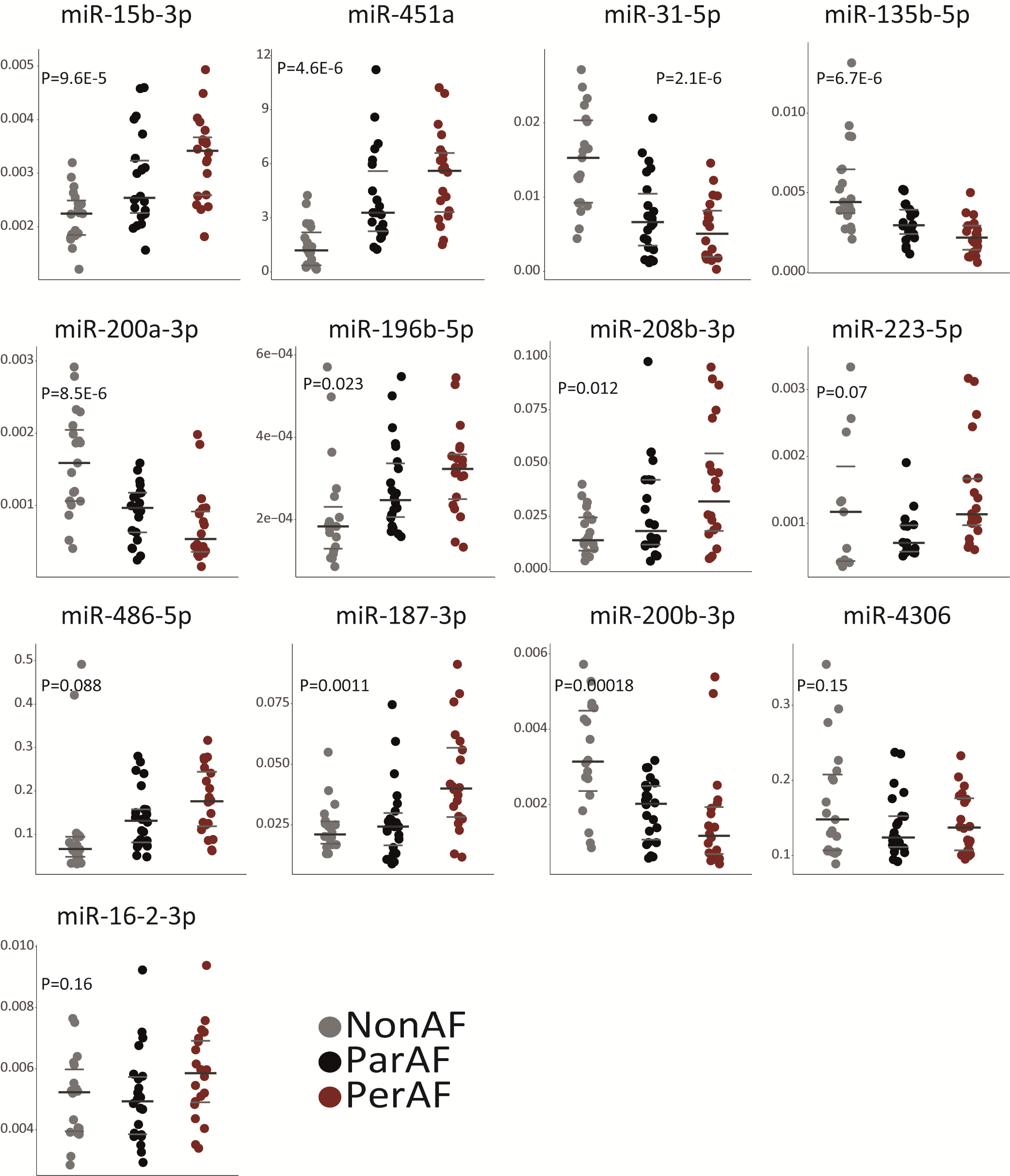
**

**Supplementary Figure S4. miRNA effects on gene expression signatures
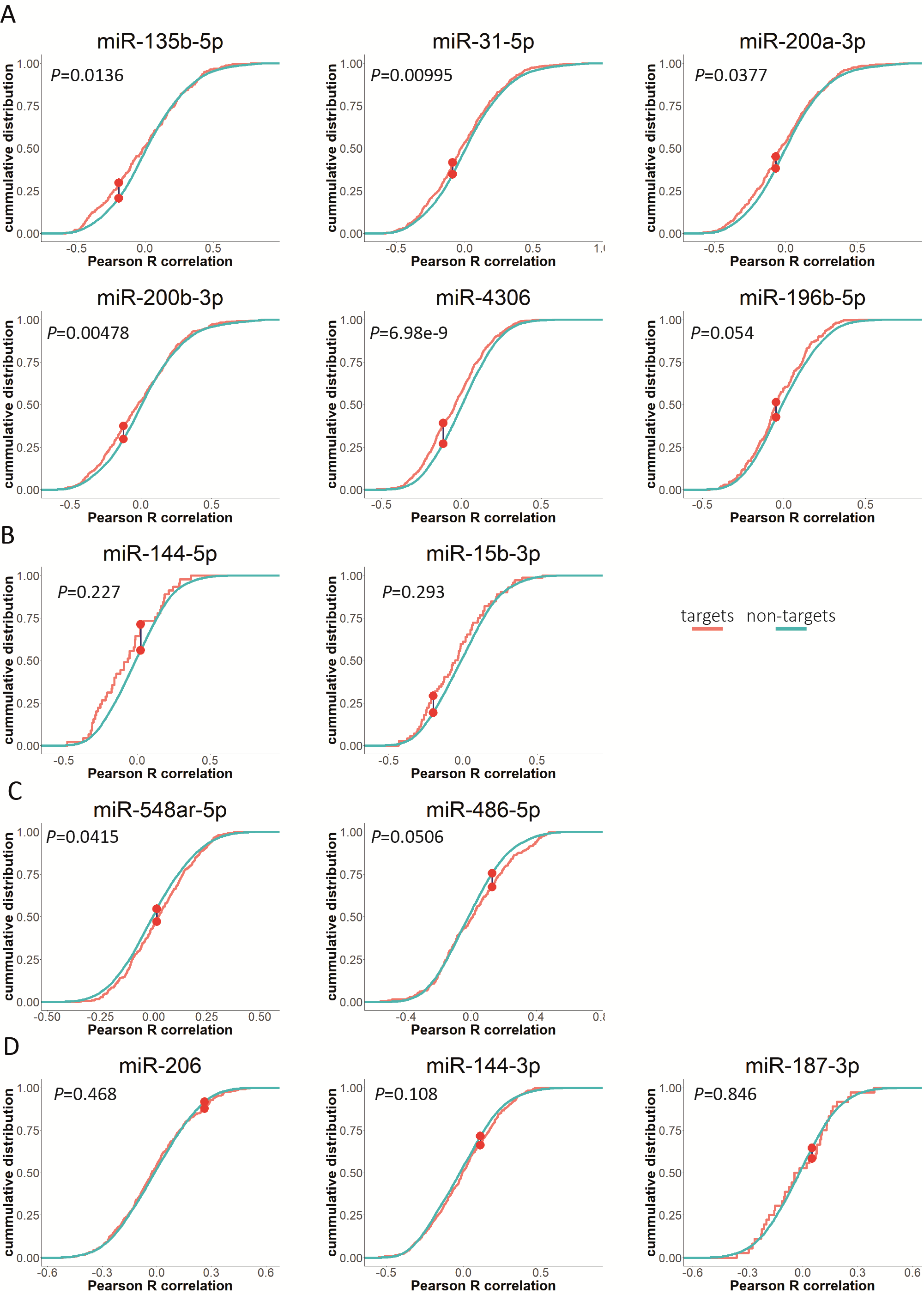
**

**Supplementary Figure S5. miRNA effects on gene expression signatures**

**
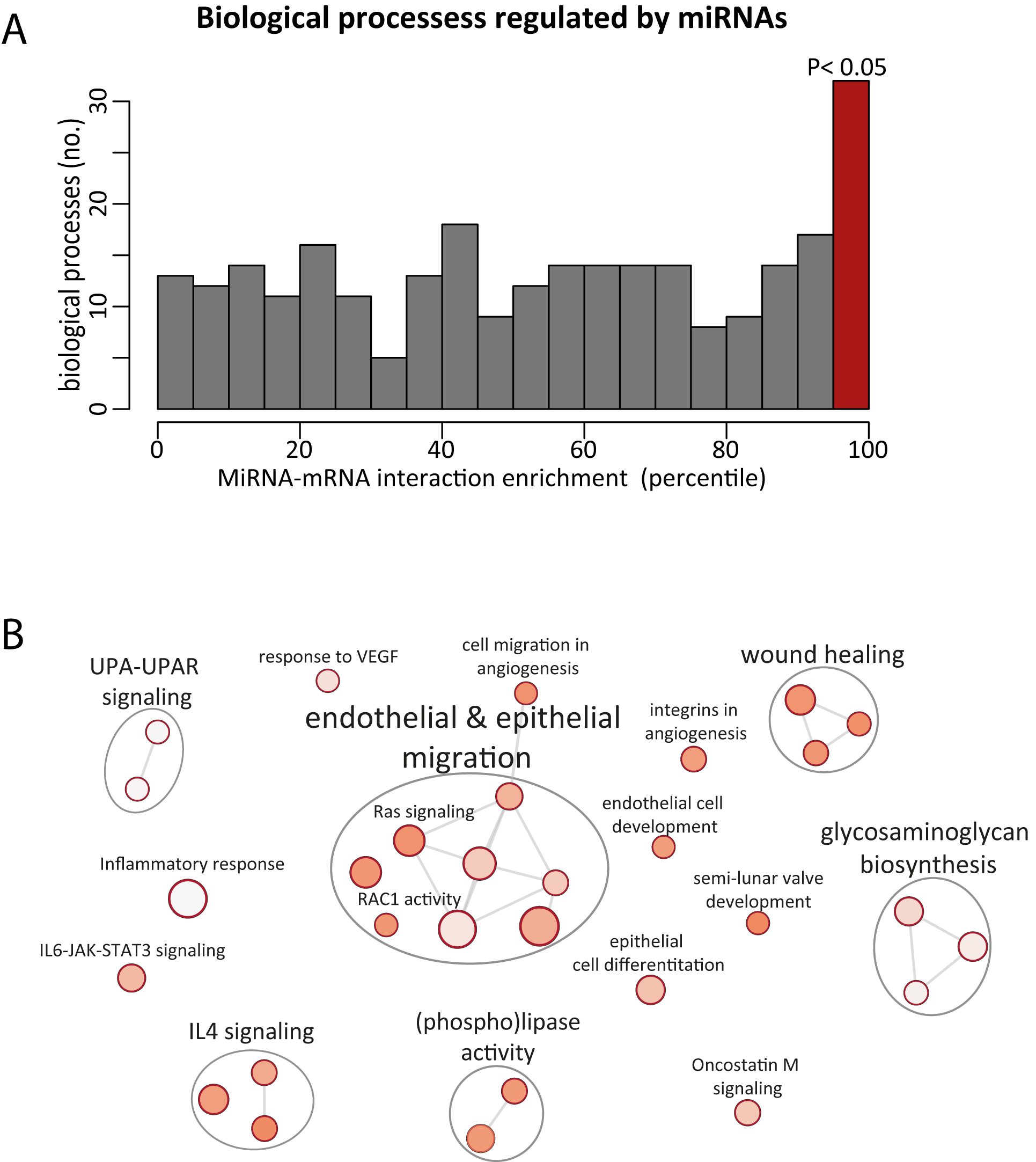
**

**Supplementary Figure S6. MiR-138-5p inhibition upregulates target genes also after exclusion of outliers**

**
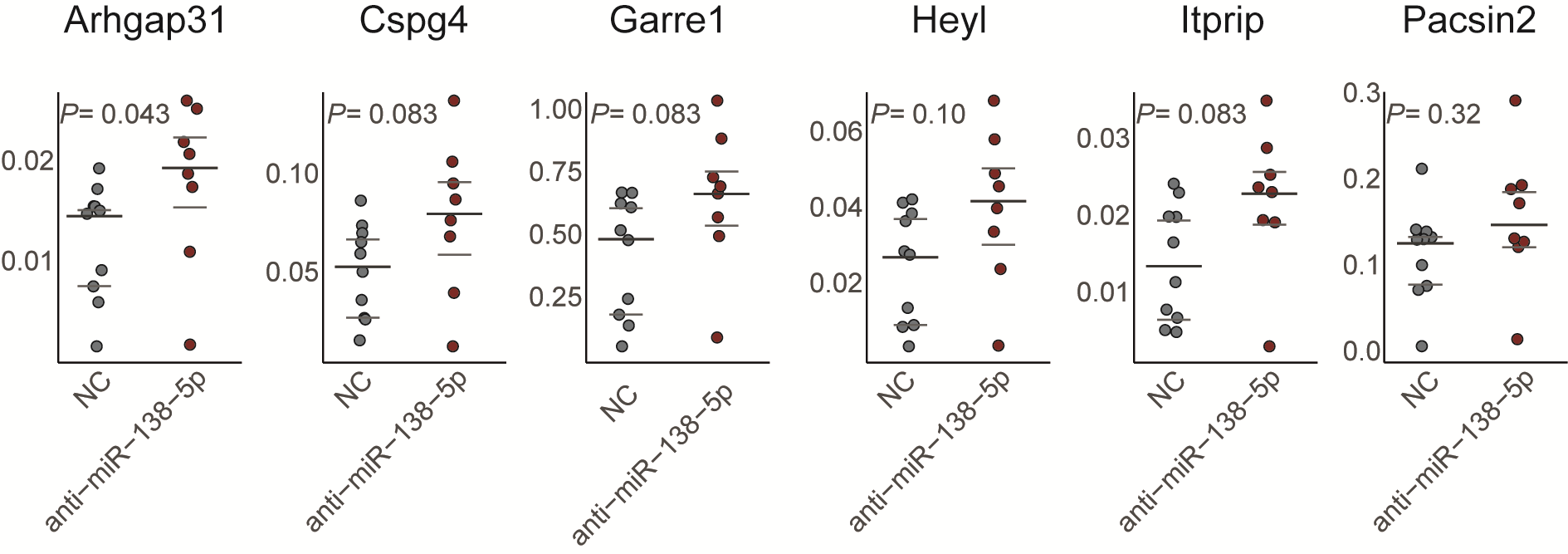
**

**Supplementary Figure legends**

**Supplementary Figure S1. Multidimensional scaling identified nonAF outlier that was subsequently removed from further analysis.**

**Supplementary Figure S2. miRNA expression validation with qPCR (continued)**

Results of miRNA qPCR validation (continued). Note that a nonAF samples appears to be an outlier. Exclusion of this outlier does not yield fundamentally different results. *P-*values in upper right or left corner represent the overall variation between the three groups and were calculated with One-way-ANOVA or Kruskal-Walllis Test when appropriate. A post-hoc analysis using a two sample t-test was performed if *P>*0.1 since some miRNAs showed group specific or mild expression changes. To avoid over-analysis, no post-hoc analyses were performed for miRNAs that demonstrated variation at *P<*0.1. Post-hoc analyses were indicated when significant. **P*<0.05; ***P*<0.01; ****P*<0.001.

**Supplementary Figure S3. miRNA expression can be validated also after exclusion of outliers.**

Results of miRNA qPCR validation after exclusion of the most extreme outlier(s). Only miRNAs with outliers are displayed. *P-*values in upper right or left corner represent the overall variation between the three groups and were calculated with One-way-ANOVA or Kruskal-Walllis Test when appropriate.

**Supplementary Figure S4. miRNA effects on gene expression signatures**

**(A-D).** Cumulative distribution functions were plotted for predicted and non-targets (CPM>3) of top differential expressed miRNAs. *P*-values were calculated using a Kolmogorov-Smirnov test. **(A.)** Top miRNAs that show more negative correlations among predicted targets than among non-targets. **(B.)** MiR-144-5p and miR-15b-3p have a lower number of predicted targets. They visually, but not statistically, demonstrate to have more negative correlations among predicted targets. **(C.)** MiR-548ar-5p and miR-486-5p have, like miR-223-3p, more positive correlations among its predicted targets. **(D.)**  MiR-206, miR-144-3p and miR-187-3p show no relation between Pearson R correlations and predicted targets.

**Supplementary Figure S5. miRNA effects on gene expression signatures**

**(A**.) Results of permutation testing for the discovery of biological processes regulated by top differentially expressed miRNAs. Thirty-two processes were predicted to be regulated by miRNAs (*P*<0.05), which is 2.4 times more than the average of 13.5 per vigintile, suggesting that the miRNA enrichment analysis is able to identify processes regulated by miRNAs. **(B).** Biological processes discovered to be regulated by miRNAs by permutation testing. All depicted processes were upregulated.

**Supplementary Figure S6. MiR-138-5p inhibition upregulates target genes also after exclusion of outliers.** After exclusion of three outliers, significance levels are lower, but 4 genes still show an upregulation after miR-138-5p inhibition with a significance of P<0.1, and all genes show an upward trend. NC, negative control; *P*-values were calculated with Mann-Whitney U test.

**REFERENCES**

1. Okada Y, Muramatsu T, Suita N, et al. Significant impact of miRNA-target gene networks on genetics of human complex traits. *Sci Rep*. 2016;6.

2. Chen Y, Wang X. MiRDB: An online database for prediction of functional microRNA targets. *Nucleic Acids Res*. 2020;48(D1):D127-D131.

3. Agarwal V, Bell GW, Nam JW, Bartel DP. Predicting effective microRNA target sites in mammalian mRNAs. *Elife*. 2015;4(AUGUST2015).

4. Paraskevopoulou MD, Georgakilas G, Kostoulas N, et al. DIANA-microT web server v5.0: service integration into miRNA functional analysis workflows. *Nucleic Acids Res*. 2013;41(Web Server issue).

5. Miranda KC, Huynh T, Tay Y, et al. A Pattern-Based Method for the Identification of MicroRNA Binding Sites and Their Corresponding Heteroduplexes. *Cell*. 2006;126(6):1203-1217.

6. Hsu S Da, Lin FM, Wu WY, et al. MiRTarBase: A database curates experimentally validated microRNA-target interactions. *Nucleic Acids Res*. 2011;39(SUPPL. 1):D163.

7. Vlachos IS, Paraskevopoulou MD, Karagkouni D, et al. DIANA-TarBase v7.0: Indexing more than half a million experimentally supported miRNA:mRNA interactions. *Nucleic Acids Res*. 2015;43(D1):D153-D159.
